# Fuzzy System-Based Target Selection for a NIR Camera-Based Gaze Tracker

**DOI:** 10.3390/s17040862

**Published:** 2017-04-14

**Authors:** Rizwan Ali Naqvi, Muhammad Arsalan, Kang Ryoung Park

**Affiliations:** Division of Electronics and Electrical Engineering, Dongguk University, 30 Pildong-ro 1-gil, Jung-gu, Seoul 100-715, Korea; rizwanali@dongguk.edu (R.A.N.); arsal@dongguk.edu (M.A.)

**Keywords:** GBI, gazing at a target to select it, fuzzy system, NIR camera-based gaze tracker

## Abstract

Gaze-based interaction (GBI) techniques have been a popular subject of research in the last few decades. Among other applications, GBI can be used by persons with disabilities to perform everyday tasks, as a game interface, and can play a pivotal role in the human computer interface (HCI) field. While gaze tracking systems have shown high accuracy in GBI, detecting a user’s gaze for target selection is a challenging problem that needs to be considered while using a gaze detection system. Past research has used the blinking of the eyes for this purpose as well as dwell time-based methods, but these techniques are either inconvenient for the user or requires a long time for target selection. Therefore, in this paper, we propose a method for fuzzy system-based target selection for near-infrared (NIR) camera-based gaze trackers. The results of experiments performed in addition to tests of the usability and on-screen keyboard use of the proposed method show that it is better than previous methods.

## 1. Introduction

The field of gaze-based interaction (GBI) has witnessed a significant growth in recent years, in response to certain long-standing challenges in gaze-tracking research. GBI helps persons with disabilities communicate with other people or devices. In 1982, Bolt first showed that the gaze can facilitate human-computer interface (HCI) implementation [1]. Using gaze input to perform tasks or computer operations eventually became popular among users with motor disabilities. Mauri et al. proposed the idea of using computer-assistive technologies, i.e., joysticks, trackballs, virtual keyboards, and eye tracking devices, to interact with computers [2]. These technologies, however, do not work well for severely disabled people who cannot use certain parts of their body. Alternative communication devices such as electromyogram (EMG), electroencephalogram (EEG), and electro-oculogram (EOG) [3,4] are better options, but are expensive and unavailable to most people. They may also be frustrating for users as they require electrodes to be attached to the body. Hence, camera-based gaze detection methods are a desirable alternative. Gaze tracking methods on the 2D monitor of a desktop computer have been widely studied [5,6,7]. However, there are some limitations to these methods, i.e., the inability to control 3D space, and significant degradation in accuracy due to variations in the Z-distance between the monitor and a user’s eyes. Therefore, to control home appliances in 3D space, a non-wearable gaze tracking system has been proposed [8]. Increasing the accuracy of gaze detection has primarily been emphasized in past studies on gaze detection, but few have tackled target selection using a detection system. To the best of our knowledge, past research used methods based on the clicks of a mouse or a keyboard, the dwell time of the gaze position, and target selection by number of eye blinks. However, these methods have limitations in terms of selection speed as well as user convenience. A detailed summary past research on target selection in gaze trackers is provided in the next section.

## 2. Related Work

To select the target of interest according to a user’s intention, approaches to locating the target of his/her gaze can be categorized into gaze-based and visual saliency-based methods. The former category includes eye blinks, dwell time, on- and off-screen buttons, keystrokes, eyebrow raises, and speech. Blinking can be used to select letters from the alphabet that can solve issues in eye typing where gaze direction is utilized to point to these letters [9]. Blinking for commands and normal blinking are usually difficult to discriminate [10]; thus, eyes that are closed for a lengthy period may be required for such discrimination, which can affect performance and the user’s convenience. The dwell time-based method seems to be a better and more natural approach compared to the blinking-based method [11]. However, for this approach, the gaze tracking system requires knowledge of the position and duration of the user’s gaze on the selected object. Past research [12,13,14] has considered methods that use the dwell time of the position of a user’s gaze. Further, an approach proposed by Huckauf et al., instead of using dwell time and blink-based selection, accomplishes target selection by using antisaccades [15]. Ware et al. proposed selecting the object of interest by fixation and subsequent saccade towards on/off buttons [16].

In past research [11,12,13,14,15,16,17,18,19,20,21,22,23], one modality has been used to point to or to select the object of interest. However, these methods typically suffer from problems whereby the object is selected every time the user looks at it, intentionally or unintentionally. This problem was first pointed out as the “Midas touch problem” [17]. It needs to be avoided, and the object of interest to a user should be discriminated from unintentionally gazes at points. The selection of the object of interest by eye blink encounters difficulty in discriminating intentional from unintentional blinks. Likewise, dwell time-based selection encounters the same type of problem, i.e., if the dwell time is very short, it faces the same problem described above: the Midas touch problem; on the other hand, if dwell time is too long, it can degrade performance as well as become tire the user [18]. A possible solution is to use graphical on/off screen buttons, but this too can be problematic, as it can interfere with the user’s intention with regard to the relevant object and deviate from the object of interest. Some past work [19,20] has proposed the use of manual inputs, such as keystrokes, combined with gaze control for pointing at and selecting objects of interest. Surakka et al. introduced the idea of a frowning face to selecting the object of interest [21]. The method based on eye blinks or eyebrow raises was proposed by Grauman et al., where these were used to point at and select the object and convey the relevant command [22]. Tuisku et al. used gazing and smiling to this end, where gazing was used to point to the object and smiling as a selection tool [23]. Although these techniques performed well for their intended purposes, they have limited accuracy and speed of selection according to the user’s intention. 

To solve the problems of single modality-based methods, multiple modality-based methods need to be explored. The authors of [24] proposed the idea of a multiple modality-based method based on pupil accommodation and dwell time. However, because they did not consider information from the monitor image (textural or high-frequency information in the area of the monitor image at which a user gazes), there is room for further improvement in detecting the user’s gaze for target selection. Furthermore, the accurate measurement of pupil accommodation and dwell time is dependent on the correct detection of pupil size and the centers of the pupil and the corneal glint. Therefore, incorrect detection can affect system performance.

Researchers have also shown considerable interest in visual attention models that can be implemented to classify of intentional and unintentional gazes [25]. Saliency-based models have been used to measure the possibility of a location to attract the observer’s attention. A saliency map can be obtained from a visual image for gaze points. Attaining visual information is a low-cost preprocessing step through which visual systems (biological or artificial) select the most noticeable information from a given scene [26]. Saliency prediction with higher accuracy has a number of applications in salient object detection, automatic vehicle guidance, scene understanding, robot navigation, specifically fast regions of interest (ROI) selection in complex visual scenes, and so on [27]. A large number of saliency-based methods for detecting intentional gazes have been proposed [28,29,30,31,32,33,34,35,36]. Most models of saliency are based on bottom-up image cues as they are biologically inspired. Many theoretical models are based on the feature integration theory proposed by Treisman and Gelade [37]. They analyzed the visual features that are combined to direct human attention over conjunction search tasks. A feed forward model to combine these features with the concept of saliency map was proposed by Koch and Ullman [38], which was implemented and verified by Itti et al. [39]. Different saliency models are based on low-, middle-, and high-level features. All models have different assumptions and methodologies focusing on different aspects of human visual behavior. These models can mainly be categorized into bottom-up, top-down, and learning-based approaches [27].

In the bottom-up approach [39,40,41,42,43,44,45], saliency models use biologically reasonable low-level features depend on computational principles proposed by Itti et al. [39]. On the basis of image features, i.e., color, intensity, and orientation, they derived bottom-up visual saliency based on center-surround differences across multi-scale image features. Bruce and Tsotsos [40] proposed attention for an information maximization (AIM) saliency model, but it is weak in detecting local dissimilarities (i.e., local vs. global saliency). A graph-based visual saliency (GBVS) model was proposed by Harel et al. based on the assumption that image patches that are different from surrounding patches are salient [41]. Like Itti et al.’s method, GBVS also fails to detect global visual saliency. Liu et al. [42] identified ROI by conditional random fields (CRF) using three types of features: multi-scale contrast, center-surround histogram, and color spatial distribution. Kong et al. proposed the idea of a multi-scale integration strategy to combine various low-level saliency features [43]. Fang et al. proposed compressed domain saliency detection using color and motion characteristics [44]. Jiang et al assumed that the area of interest of visual attention has a shape prior, and detected salient regions using contour energy computation [45]. Low-level feature-based methods perform well in general but do not consider semantic information, such as faces, humans, animals, objects, and text.

To solve this problem, the top-down approach has been researched [46,47,48,49]. Several studies have adopted this approach and attained performance improvement by adding high-level features. Cerf et al. [46] added a high-level factor, face detection, to Itti’s model and showed that it enhances performance. Judd et al. [47] proposed a saliency detection model by learning the best weights for all combined features using support vector machines (SVMs). Chang et al. [48] subsequently proposed the idea of an object-based saliency model by relying on the claim that observers tend to look at the centers of objects. Hence, top-down models have highlighted the importance of high-level and semantic features, e.g., faces, animals, the image-centric bias, and the object-centric bias. A major problem with this approach is that it is training dependent, and often fails to detect salient features if its models are not trained. To consider the limitations of the bottom-up and top-down approaches, the learning-based method has been researched [50,51,52,53,54,55,56,57]. An AdaBoost-based model for feature selection was proposed by Borji [52]. It models complex input data by combining a series of base classifiers. Reingal et al. observed that there were different statistics for fixated patches compared to random patches [53]. Borji et al. [56] later combined two simple methods (multiplication and sum) using normalization schemes (identity, exponential, and logarithmic) to combine saliency models. The evolutionary optimization algorithm has been used by some researchers to find optimal sets of combination weights [57].

All saliency-based methods can provide only information concerning regions where people tend to gaze with higher probability, instead of accurate intentional gaze position. Therefore, to overcome the limitations of past work on gaze-based methods as well as visual saliency-based methods, we propose a fuzzy system-based target selection method for near-infrared (NIR) camera-based gaze trackers by fusing the gaze-based method with the bottom-up visual saliency-based method. Our method combines multi-modal inputs, i.e., pupil accommodation measured by template matching, short dwell time, and Gabor filtering-based texture information of visual saliency using a fuzzy system. In the following four ways, our research is novel compared to past research. The first, second, and third points represent major novelties whereas the fourth is a minor one:

- First, a new and improved method based on the Chan–Vese algorithm is proposed for detecting the pupil center with a boundary as well as the glint center.
- Second, we use three features, i.e., change in pupil size using template matching (to measure pupil accommodation), change in gaze position during short dwell time, and Gabor filtering-based texture information of monitor image at gaze target. A fuzzy system is then used with these three features as inputs, and the decision concerning the user’s target selection is taken through defuzzification.
- Third, an optimal input membership function for fuzzy system can be obtained based on the maximum entropy criterion.
- Fourth, through comparative experiments using an on-screen keyboard on a previous dwell time-based method, the performance and usability of our method were verified in a real gaze-tracking environment.

In Table 1, we summarize the comparison of the proposed with existing methods.

The remainder of this paper is organized as follows: in Section 3, our proposed system and methodology are introduced. The experimental setup is explained and the results are presented in Section 4. Section 5 contains our conclusions and discussion of some ideas for future work.

## 3. Target Selection by Using Pupil Accommodation with Template Matching, Change in Gaze, and Texture Information from a Monitor Image of Gaze Target

### 3.1. Overview of Proposed Method

In the proposed method, images of the eye were taken using an image acquisition system on a commercial Web camera, i.e., a Logitech (Lausanne, Switzerland) C600 [58] with a universal serial bus (USB) interface, and near-infrared (NIR) was used as source of illumination, with 8 × 8 NIR light-emitting diodes (LEDs) for our gaze tracker. The use of NIR illuminator served three important purposes [59,60]: first, it minimized the impact of different ambient light conditions; second, it distinguished the boundary of the pupil; third, as NIR is barely visible, it can minimize any interference while using the device in applications. In detail, the NIR light of shorter wavelength (shorter than 800 nm) has the tendency of making the iris darker (compared to the case using NIR light of wavelength greater than 800 nm). Therefore, the boundary between the pupil and the iris in the image becomes less distinct, and the error associated with locating the pupil boundary increases. On the other hand, NIR light of longer wavelength (longer than 800 nm) has the opposite tendency of making the iris brighter. Therefore, the boundary between the pupil and the iris in the image becomes more distinct, and the error associated with locating the pupil boundary is reduced. However, the camera image sensor is usually less sensitive to light according to the increase in wavelength, which means that the image captured by light of wavelength longer than 900 nm becomes darker, and the correct detection of the pupil boundary is consequently difficult. By considering all these factors, an NIR illuminator of 850 nm was adopted in our gaze tracking system.

Images of 1600 × 1200 pixels were captured at a rate of 30 frames per second (fps). Larger eye images were required to analyze pupil size variations during the user’s gaze for target selection. Thus, we used a zoom lens to obtain these images. For pupil accommodation, we need a gaze tracking system that can measure changes in pupil size. Unfortunately, not all commercial gaze tracking systems provide this function [61,62,63,64]. Therefore, we implemented our own system.

**Figure 1:**
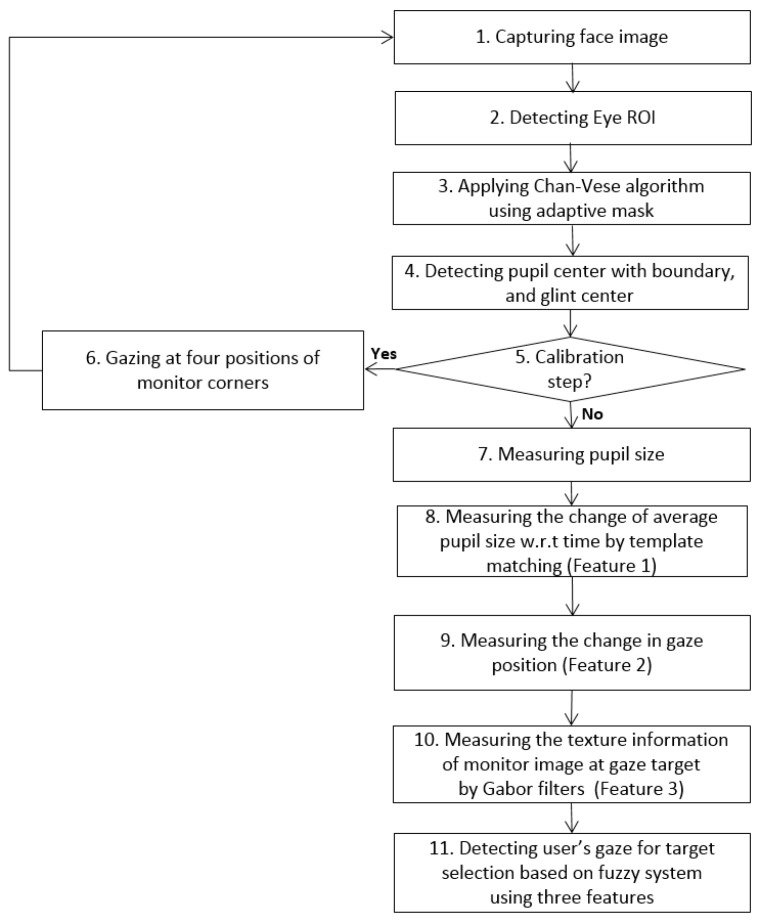
Overall procedure of the proposed method.

**Figure 2:**
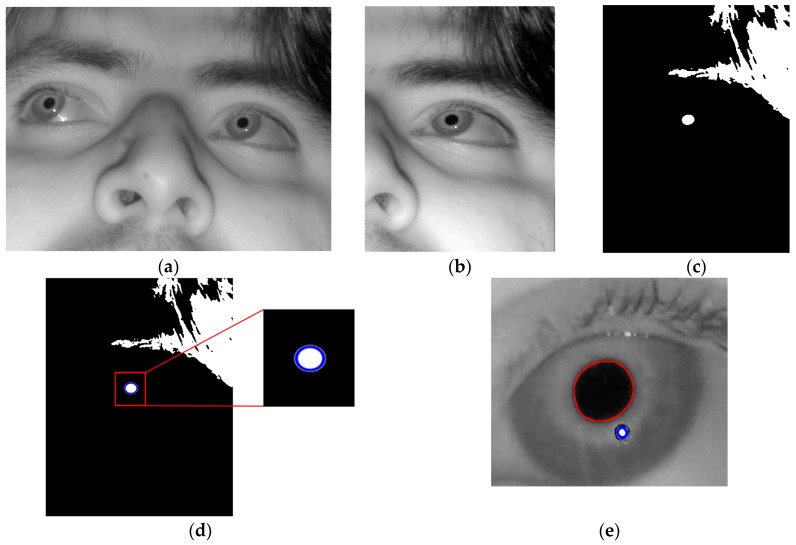
Example of detecting pupil and glint of right eye. (**a**) Input face image; (**b**) Search area of right eye; (**c**) Binarized image; (**d**) Approximate pupil area detected by CHT, and eye ROI defined on the basis of approximate pupil area; (**e**) Pupil and glint boundaries detected by Chan–Vese algorithm with adaptive mask.

Figure 1 shows the flowchart of our proposed system. An image of the user’s face is first captured by our gaze tracking camera, and the search area of the left or right eye is defined (as shown in Figure 2b). Within this area, the pupil center with boundary and the glint center are found (see the details in Section 3.2). The bright spot on the corneal surface caused by the NIR light is referred to as glint. In the initial step, the user is instructed to observe four positions on the monitor for user-dependent calibration. Pupil size is then measured based on the accurate boundary of the pupil region (see the details in Section 3.3). The features are then calculated to detect the user’s gaze for target selection. Feature 1 (*F*_1_) represents pupil accommodation, i.e., it is measured by template matching with the graph of change in pupil size with respect to time (see the details in Section 3.3). The change in gaze position calculated over a short dwell time is referred to as feature 2 (*F*_2_) (see the details in Section 3.4). Furthermore, the texture information of the image on the monitor at the gaze target is measured by Gabor filters and is used as feature 3 (*F*_3_) (see the details in Section 3.5). These three feature values are combined using a fuzzy system, and the user’s gaze for target selection can be detected based on the fuzzy output (see the details in Section 3.6).

### 3.2. Detection of Pupil and Glint Centers

**Figure 3:**
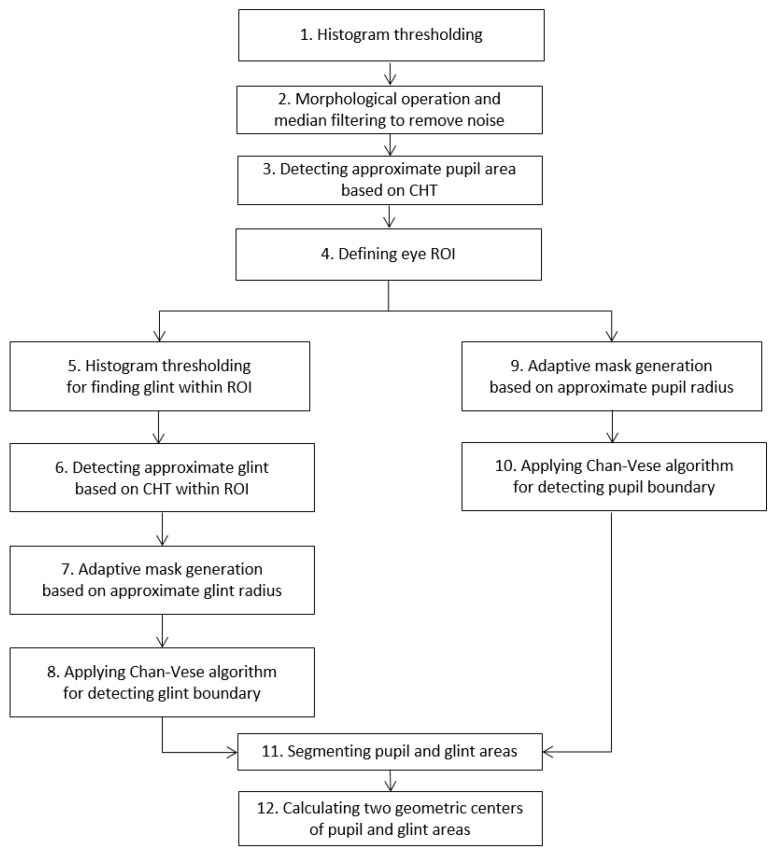
Flowchart for detecting glint center and pupil region.

The correct detection of the pupil region and the glint center is a prerequisite for obtaining accurate feature values for detecting the user’s gaze for target selection. In our research, the pupil and glint regions (with geometric centers) were detected as shown in Figure 3, which corresponds to Steps 2–4 of Figure 1.

The approximate pupil area is first extracted as follows: to find dark pixels in the acquired image, binarization based on histogram thresholding is performed (Step 1 in Figure 3). Morphological operations and media filtering operations are executed to remove noise (Step 2 in Figure 3), and the approximate pupil area is detected (Step 3 of Figure 3) based on the circular Hough transform (CHT) [65]. The CHT is a basic technique for detecting circular objects in an image. The concepts of voting and local maxima, i.e., an accumulator matrix, are used to select candidate circles. The ROI including the eye is defined in the input image on the basis of the approximated pupil region (Step 4 of Figure 3). The accurate boundaries of the pupil and the glint are then located within the ROI using the Chan–Vese algorithm [66] based on an adaptive mask (Steps 5–10 of Figure 3). It depends on global properties, i.e., gray-level intensities, contour lengths, and regional areas, rather than local properties such as gradients. The main idea behind the Chan–Vese algorithm is active contour models to evolve a curve from a given image $u_{o}$ to detect objects in the image. In the classical active contour and snake models [67], an edge detector is used that depends on the gradient of image$\;u_{o}$ to stop the evolving curve on the boundary of the required object. By contrast, in the Chan–Vese algorithm, the model is based on trying to separate the image into regions based on intensities. We minimize the fitting terms and add some regularizing terms like the length of the curve *C* and/or the area of the region inside *C*. We want to minimize the energy function using level set ϕ(x,y) formulation [66]:

(1) C=∂ω={(x,y)ϵ Ω: ϕ(x,y)=0}cin=ω={(x,y)ϵ Ω: ϕ(x,y)>0}cout=Ωω¯={(x,y)ϵ Ω: ϕ(x,y)<0}

where C=∂ω is the curve where ω ϵ Ω, and Ω is the planar domain. cin=ω, cout=Ωω¯ represent regions inside and outside curve C, respectively. The energy function *F*($c_{in},\;c_{out},\;C$), is defined by:

(2) $$F\left(c_{in},\;c_{out},\;C\right)=\mu \cdot Length\left(C\right)+\;\lambda_{in}{\int^{\text{​}}}_{in\left(C\right)}{\left|u_{o}\left(x,y\right)-c_{in}\right|}^{2}dxdy+\lambda_{out}{\int^{\text{​}}}_{out\left(C\right)}{\left|u_{o}\left(x,y\right)-c_{out}\right|}^{2}dxdy$$

In the above, $\lambda_{in},\;\lambda_{out},\;\mathrm{and}\;\mu \;$are the positive constants, and $u_{o}\left(x,y\right)$ is the given input image. In order to enhance processing speed and accuracy, we propose an enhanced Chan–Vese algorithm with an adaptive mask based on the approximate pupil region (Steps 9 and 10 of Figure 3) (we use the constraint where (*x*, *y*) of Equation (2) belongs to the approximated pupil area). The mask parameters change according to pupil size and location. The CHT provides the rough radius and center of the pupil area of Step 3 in Figure 3. Based on this information, an accurate pupil boundary can be obtained by the Chan–Vese algorithm with adaptive mask, as shown in Steps 10 and 11 of Figure 3. Moreover, the rough size and location of the glint are detected by histogram thresholding and CHT, as shown in Steps 5 and 6 of Figure 3. Based on this information, an accurate glint boundary can be obtained by the Chan–Vese algorithm with adaptive mask as shown in Steps 8 and 11 of Figure 3 (we use the constraint whereby (*x*, *y*) of Equation (2) belongs to the approximated glint area). Using the boundaries of the pupil and the glint, their geometric centers are determined as shown in Step 12 of Figure 3.

Figure 2 shows the resulting images of the procedure. Based on these detection results of the pupil and glint areas, two features for inputs to the fuzzy system are calculated, as shown in Section 3.3 and Section 3.4.

### 3.3. Calculating Feature 1 (Change in Pupil Size w.r.t. Time)

As the first feature used to detect the user’s gaze for target selection, the change in pupil size with respect to time is measured by template matching. The authors of [24] measured pupil size based on the lengths of the major and minor axes of an ellipse fitted along the pupil boundary. The actual shape of the pupil is not elliptical, as shown in Figure 2e, and, according to [68], pupil detection based on ellipse fitting can yield incorrect pupil size. Therefore, in our research, pupil size is measured by counting total number of pixels inside the pupil boundary detected by the enhanced Chan–Vese method. A graph representing the in pupil size with respect to time is then constructed, and a moving average filter consisting of three coefficients is applied to it to reduce noise.

As mentioned above, the speed of image acquisition of our gaze tracking camera was 30 frames per second. Hence, an image frame was captured in 33.3 ms (1/30 s). To increase the speed of target selection, we used a window of size 10 frames (approximately 333 ms) to measure pupil dilation and constriction over time. Even with a short time window, our method can detect the user’s gaze for target selection because three features are simultaneously used.

**Figure 4:**
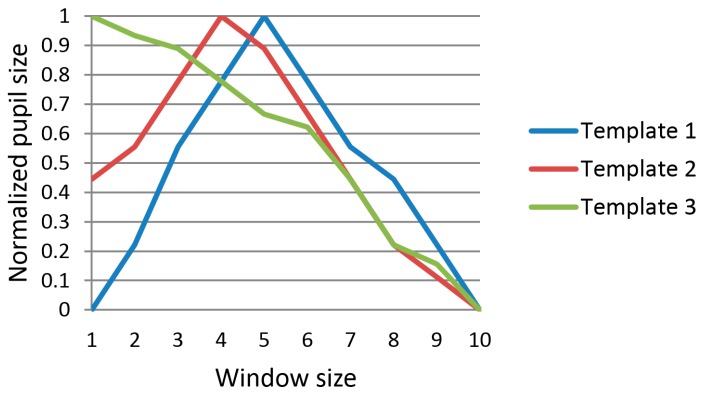
Three template graphs for calculating template matching score.

Past research has shown that cognitive tasks can affect changes in pupil size [69,70]. Based on this concept, the size of the pupil usually decreases in case the gaze is used for target selection, and we can define the shapes resulting from changes in pupil size as shown in Figure 4. Using these shapes, template-based matching is performed with a given window size. Template-based matching is the measure of the similarity between the graph of dilation and constriction in pupil size with respect to time and the expected graph of pupil behavior (template graph shown in Figure 4) employing the user’s gaze for target selection. Template-based matching compares these two graphs using the sum of square differences (SSD). The SSD and the final template matching score can be obtained by the following equations:

(3) $$\mathrm{SSD}(p_{j},q_{ij})=\;\sum_{j=1}^{10}{\left(p_{j}-q_{ij}\right)}^{2}$$

(4) $$\text{Template matching score}\;=\underset{i=1,\;2,\;3}{\min}\mathrm{SSD}\left(p_{j},q_{ij}\right)$$

In the above, *p_j_* is the *j*th value of pupil size in the graph of the input, and *q_ij_* is the *j*th value of the pupil size of the *i*th template graph. We used three template graphs as shown in Figure 4. The starting position of the template matching window was detected based on changes in gaze position, i.e., change in the horizontal and vertical gaze directions. The template matching score of Equation (1) decreased in case the user’s gaze was employed for target selection and increased in other cases. The template matching score was used as feature 1. Since the maximum and minimum values of pupil size can show individual variation according to people, the value of pupil size was normalized by min-max scaling before template matching, as shown in Figure 4. Although one previous study [24] used a similar concept to measure the change in pupil size through peakedness, this method has the disadvantage whereby the accurate position of the peak needs to be detected in advance in the graph for pupil size, which can be affected by noise in the input data. In contrast to this, our method does not need to detect the position of the peak, and is more robust against noise in the input data (see Section 4).

### 3.4. Calculating Feature 2 (Change in Gaze Position within Short Dwell Time)

**Figure 5:**
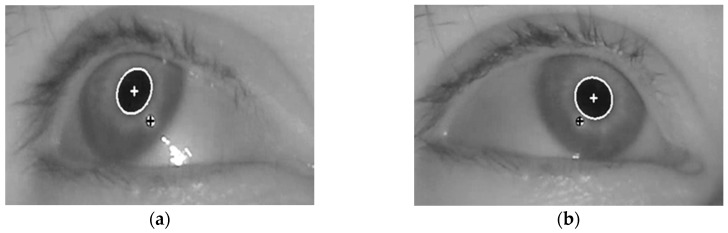

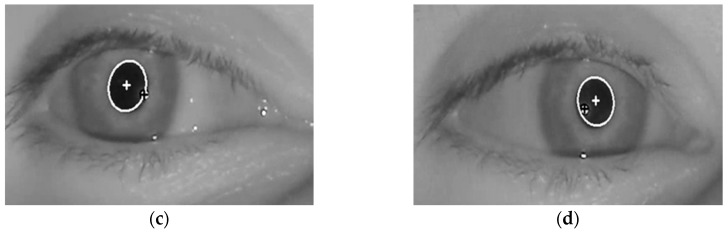
Four images showing the detected centers of glint and the pupil, when a person is looking at the (**a**) upper-left; (**b**) upper-right; (**c**) lower-left; and (**d**) lower-right calibration positions on a monitor.

The detected pupil center and glint center (explained in Section 3.2) are used to calculate the gaze position, i.e., feature 2. Initial user calibration is performed to calculate gaze position. For calibration, each user is instructed to examine four positions close to the corners of the monitor [24]. From this, four pairs of pupil centers and glint centers are obtained, as shown in Figure 5.

**Figure 6:**
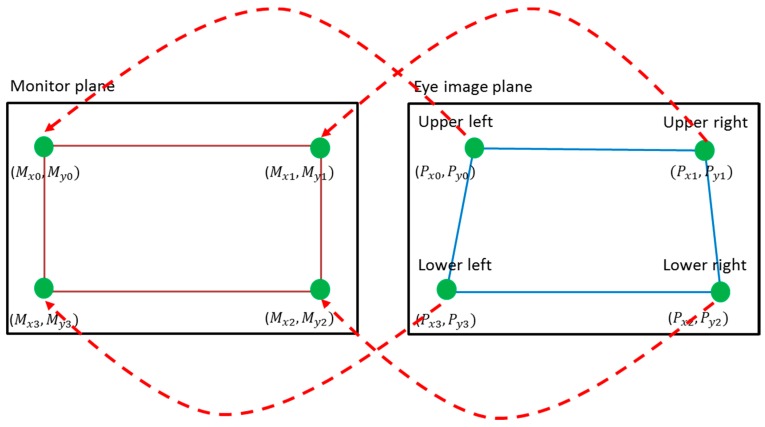
Relationship between pupil movable region (the quadrangle defined by $\left(P_{x0},P_{y0}\right)$, $\left(P_{x1},P_{y1}\right)$, $\left(P_{x2},P_{y2}\right)$, and $\left(P_{x3},P_{y3}\right)$) in the eye image and the monitor region (the quadrangle defined by $\left(M_{x0},M_{y0}\right)$, $\left(M_{x1},M_{y1}\right)$, $\left(M_{x2},M_{y2}\right)$, and $\left(M_{x3},M_{y3}\right)$).

The position of the center of the pupil is compensated for by that of the glint center, which can reduce the effect of head movement on the variation in gaze position. A geometric transform matrix can be calculated with these four pairs of detected pupil centers and glint centers [24]. This matrix defines the relationship between the region occupied by the monitor and that by the movable pupil, as shown in Figure 6.

Then, geometric transform matrix is calculated using Equation (5), and the position of the user’s gaze (*G_x_*, *G_y_*) is calculated by Equation (6):

(5) $$\left[\begin{array}{c} M_{x0}\;M_{x1}\;M_{x2}\;M_{x3}\\ M_{y0}\;M_{y1}\;M_{y2}\;M_{y3} \end{array}\right]=\left[\begin{array}{cc} \begin{array}{cc} a & b\\ e & f \end{array} & \begin{array}{cc} c & d\\ g & h \end{array} \end{array}\right]\left[\begin{array}{cccc} P_{x0} & P_{x1} & P_{x2} & P_{x3}\\ P_{y0} & P_{y1} & P_{y2} & P_{y3}\\ P_{x0}P_{y0} & P_{x1}P_{y1} & P_{x2}P_{y2} & P_{x3}P_{y3}\\ 1 & 1 & 1 & 1 \end{array}\right]$$

(6) $$\left[\begin{array}{c} G_{x}\\ G_{y} \end{array}\right]=\left[\begin{array}{cccc} a & b & c & d\\ e & f & g & h \end{array}\right]\left[\begin{array}{c} P\prime_{x}\\ P\prime_{y}\\ P\prime_{x}P\prime_{y}\\ 1 \end{array}\right]$$

As the two positions are obtained from the left and the right eyes, the final gaze position is calculated by taking the average of these two gaze positions. Gaze position is not usually moved in case of the user’s gaze for target selection. Therefore, the Euclidean distance ($\Delta z_{i}$) of gaze positions between the given ($x_{i}$,$\;y_{i}$) and the previous image frames ($x_{i-1}$,$\;y_{i-1}$) is calculated as shown in the Equation (7). Then, feature 2 (change in gaze position within a short dwell time) is calculated from the estimated starting (time) position of the gaze, i.e., *S* of Equation (8), over a specified short dwell time (the window size *W* of Equation (8)), and feature 2 becomes smaller in case of the employment of the user’s gaze for target selection:

(7) $$\Delta z_{i}=\sqrt{{\left(x_{i}-x_{i-1}\right)}^{2}+{\left(y_{i}-y_{i-1}\right)}^{2}}$$

(8) $$\mathrm{Feature}\;2\;\left(\mathrm{change}\;\mathrm{in}\;\mathrm{gaze}\;\mathrm{position}\;\mathrm{within}\;\mathrm{short}\;\mathrm{dwell}\;\mathrm{time}\right)=\overset{W+S-1}{\underset{i=S}{\sum }}\Delta z_{i}$$

Although a previous study [24] used a similar concept to measure changes in gaze position, this method involves measuring the change in gaze position by selecting the larger of two changes in gaze position along the horizontal and the vertical directions. For example, if the changes in gaze position are, respectively, 3 and 4 along the horizontal and vertical directions, feature 2 measured by this method is 4 instead of 5 ($\sqrt{3^{2}+4^{2}}$). By contrast, our method measures the change in gaze position both along the horizontal and the vertical directions, and feature 2 is measured as 5 in this case.

### 3.5. Calculating Feature 3 (the Texture Information of Monitor Image at Gaze Target)

**Figure 7:**
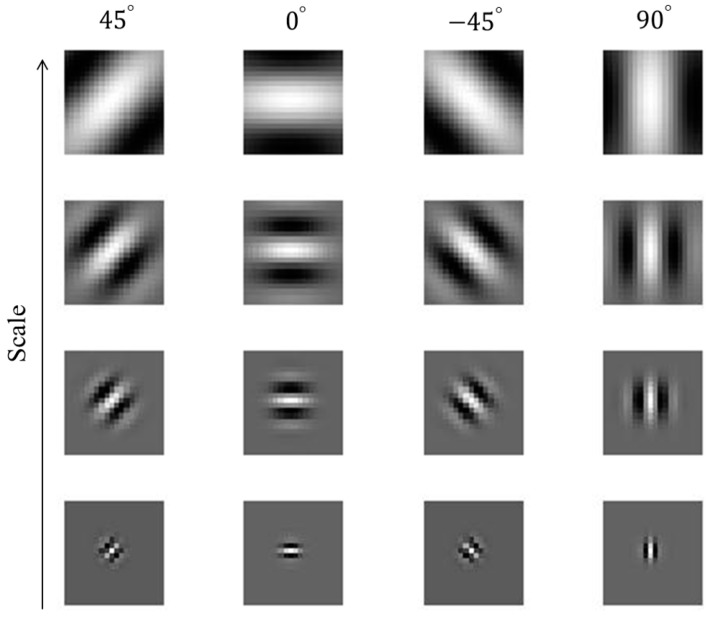
16 Gabor filters with four scales and four orientations.

**Figure 8:**
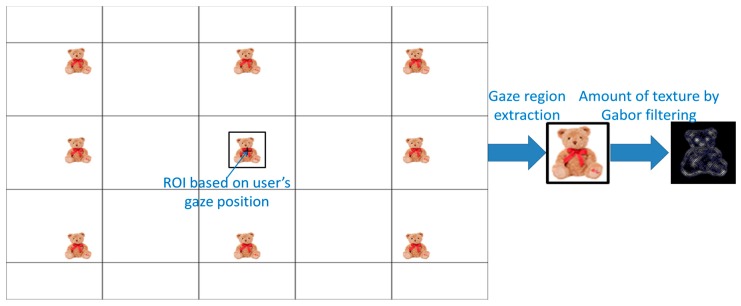
ROI for applying Gabor filter based on the user’s gaze position.

Baddeley et al. and Yanulevskaya et al. found that edge frequencies are strong contributors to the location of the user’s gaze, and have higher correlations to it than other factors [71,72]. Based on this concept, we extract edge-based texture information from the expected gazing location using a Gabor filter [73], and use this information as feature 3 to detect the user’s gaze for target selection. The region where the object of interest is located has a larger amount of texture than where it is not. A 2D Gabor filter in the spatial domain is defined as follows:

(9) $$g\left(x,y\right)=\left(\frac{1}{2\pi \sigma_{x}\sigma_{y}}\right)exp\left[-\frac{1}{2}\left(\frac{x^{2}}{\sigma_{x}{}^{2}}+\frac{y^{2}}{\sigma_{y}{}^{2}}\right)+2\pi jWx\right]$$

where g(x,y) is defined as the Gabor function along the *x*- and *y*-axes. $\sigma_{x}\;$and $\sigma_{y}$ are standard deviations of the function along the *x*- and *y*-axes, respectively. *W* is the radial frequency of a sinusoid. We consider only the real part of the Gabor filter for fast processing. To obtain the Gabor wavelet, g(x,y) in Equation (9) is used as the mother Gabor wavelet. The Gabor wavelet is then obtained by the scaling and rotation of g(x,y) as shown in Equation (10):

(10) $$\;g_{s}\left(x,y\right)=\;a^{-m}g\left(x^{\prime },y^{\prime }\right),\;(x^{\prime }=xcos\theta +ysin\theta ,\;y^{\prime }=-xsin\theta +ycos\theta )$$

where θ is the filter orientation expressed by θ = *nπ*/*K*, *K* is the number of the filter orientations (*n* is the integer number), $a^{-m}$ is the filter scale, and *m* = 0, …, *P*, *P* + 1 is the number of scales. In order to extract the accurate texture, we use 16 Gabor wavelet filters with *K* = 4 and *P* = 3, as shown in Figure 7. The average number of magnitudes obtained using Gabor wavelet filters within the ROI is used as feature 3. For this, the ROI for the application of the Gabor filter is defined based on the user’s gaze as shown in Figure 8. According to the gaze position, the magnitude of texture in the ROI varies, and the user’s gaze shows a high value for feature 3, due to the complex texture of the monitor image.

### 3.6. Fuzzy Logic System for Detecting User’s Gaze for Target Selection

#### 3.6.1. Explanation of Fuzzy Membership Functions

**Figure 9:**
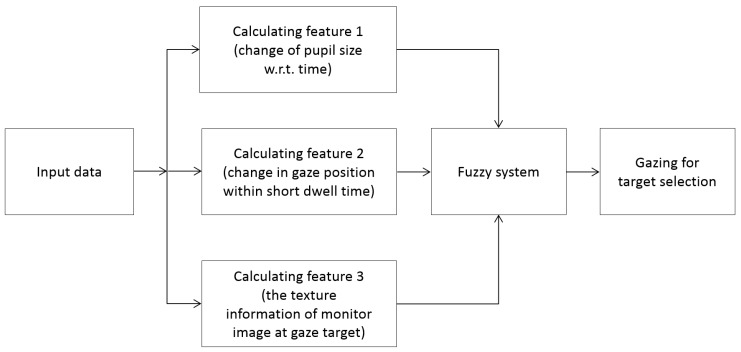
Fuzzy method for detecting user’s gaze for target selection.

To detect the user’s gaze for target selection, our method uses a fuzzy system with three input features, i.e., features 1–3 (explained in Section 3.3, Section 3.4 and Section 3.5), as shown in Figure 9. As explained in Section 3.3, Section 3.4 and Section 3.5, features 1 and 2 are smaller, whereas feature 3 is larger in case the user’s gaze is used. Through normalization based on min-max scaling, these three features are made to range from 0 to 1. To make these three features consistent with one another, features 1 and 2 are recalculated by subtracting them from the maximum value (1). Therefore, features 1–3 are larger in when the user’s gaze is used, and are used as inputs to the fuzzy logic system. Based on the output of the fuzzy system, we can determine whether the user is gazing at the selected target.

**Figure 10:**
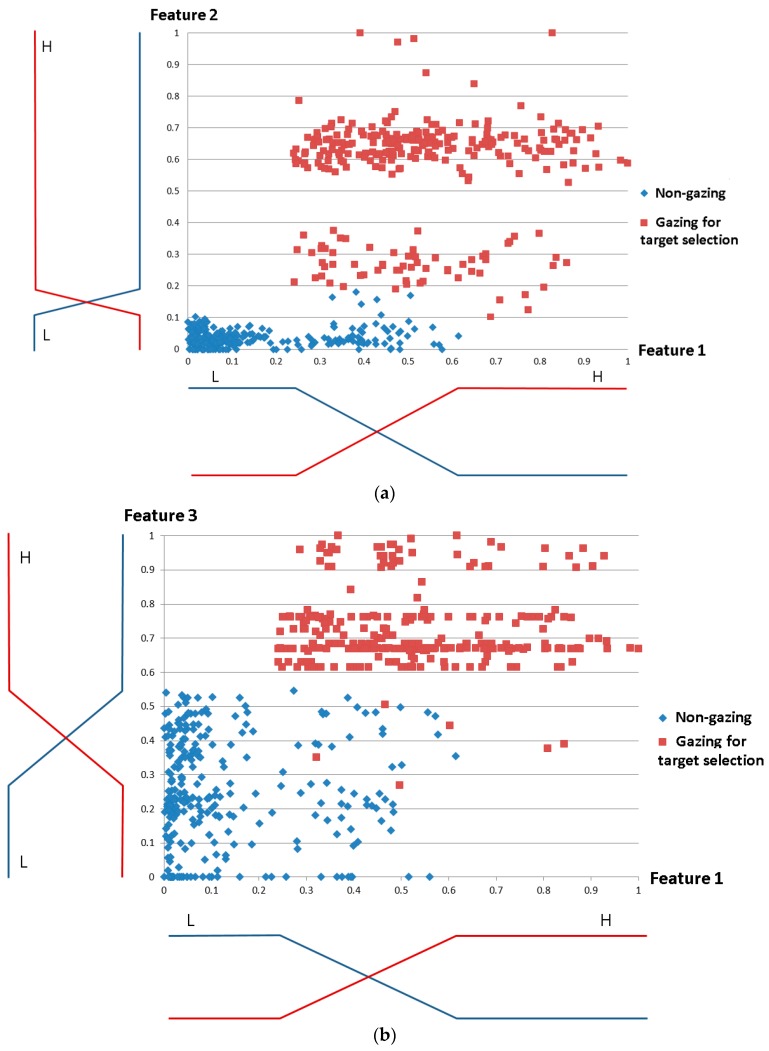
Designing optimal input fuzzy membership functions for features 1–3 based on maximum entropy criterion. (**a**) Membership functions for features 1 and 2; (**b**) Membership functions for features 1 and 3.

Figure 10 shows the membership functions for the three input features 1–3. The input values were classified into two classes in the membership function: Low (L) and High (H). In general, these value classes are not separated, and the membership functions are defined to have overlapping areas, as shown in Figure 10. With a small number of input data items, we obtained the distributions of features 1–3 as shown in Figure 10 and, based on the maximum entropy criterion [74], designed the input membership functions. For fair experiments, these data were not used for all experiments reported in Section 4.

We first define the rough shape of the input membership functions as linear by considering processing speed and the complexity of problem because such functions have been widely used in fuzzy applications [75,76,77]. The defined input membership functions are as follows:

(11) $$\mu_{L\_feature\;i}\left(x\right)=\left\{\;\begin{array}{c} \begin{array}{cc} 1 & \mathrm{for}\;0\leq x\leq a_{L\_i}\\ p_{L\_i}x+q_{L\_i} & \mathrm{for}\;a_{L\_i}\leq x\leq b_{L\_i}\\ 0 & \mathrm{for}\;b_{L\_i}\leq x\leq 1 \end{array} \end{array}\right\}$$

(12) $$\mu_{H\_feature\;i}\left(x\right)=\left\{\;\begin{array}{c} \begin{array}{cc} 0 & \mathrm{for}\;0\leq x\leq a_{H\_i}\\ p_{H\_i}x+q_{H\_i} & \mathrm{for}\;a_{H\_i}\leq x\leq b_{H\_i}\\ 1 & \mathrm{for}\;b_{H\_i}\leq x\leq 1 \end{array} \end{array}\right\}$$

In Equations (11) and (12), *i* = 1, 2, and 3. $\mu_{L\_feature\;i}\left(x\right)$ is the L membership function of feature i whereas $\mu_{H\_feature\;i}\left(x\right)$ is its H membership function. Then, we can obtain the following equations:

(13) $$p_{L\_feature\;i}=\;\sum_{x=0}^{1}m_{L\_feature\;i}(x)\mu_{L\_feature\;i}\left(x\right)$$

(14) $$p_{H\_feature\;i}=\;\sum_{x=0}^{1}m_{H\_feature\;i}(x)\mu_{H\_feature\;i}\left(x\right)$$

In Equations (13) and (14), *i* = 1, 2, and 3. $\mu_{L\_feature\;i}\left(x\right)$ is the L membership function of feature i of Equation (11), whereas $\mu_{H\_feature\;i}\left(x\right)$ is the H membership function of feature i in Equation (12). In addition, $m_{L\_feature\;i}\left(x\right)$ is the L (data) distribution of feature i (non-gazing data of Figure 10), whereas $m_{H\_feature\;i}\left(x\right)$ is the H (data) distribution of feature i (gazing data for target selection of Figure 10). Based on Equations (13) and (14), the entropy can be calculated as follows:

(15) $$H\left(a_{L\_i},b_{L\_i},\;p_{L\_i},\;q_{L\_i},\;a_{H\_i},b_{H\_i},\;p_{H\_i},\;q_{H\_i}\right)\;=\;-p_{L_{feature}i}\log\left(p_{L_{feature}i}\right)-p_{H_{feature}i}\log\left(p_{H_{feature}i}\right)$$

where *i* = 1, 2, and 3. Based on the maximum entropy criterion [74], the optimal parameters of $\left(a_{L\_i},b_{L\_i},\;p_{L\_i},\;q_{L\_i},\;a_{H\_i},b_{H\_i},\;p_{H\_i},\;q_{H\_i}\right)\;$of feature i are obtained by being chosen when the entropy $H\left(a_{L\_i},b_{L\_i},\;p_{L\_i},\;q_{L\_i},\;a_{H\_i},b_{H\_i},\;p_{H\_i},\;q_{H\_i}\right)\;$is maximized. From this, we can obtain the input membership functions of features 1–3.

**Figure 11:**
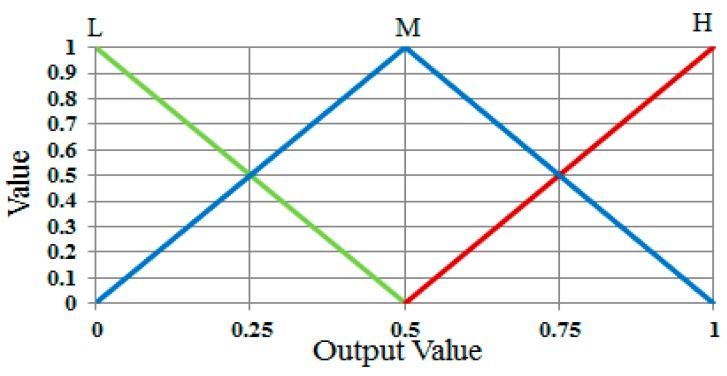
Definitions of output membership functions.

These membership functions are used to convert input values into degrees of membership. In order to determine whether the user’s gaze for target selection occurs, the output value is also described in the form of a linear function from the membership functions, as in Figure 11 that shows the three functions of L, M, and H. Using these output membership functions, the fuzzy rule table, and a combination of the defuzzification method with the MIN and MAX rules, the optimal output value can be obtained (see details in Section 3.6.3)

#### 3.6.2. Fuzzy Rules Based on Three Input Values

As explained in Section 3.6.1 and Figure 10, the values of features 1–3 are larger in case the user’s gaze is employed for target selection. We define the output of our fuzzy system as “H” in case it is, and as “L” when it is not. Based on this, we define the fuzzy rules shown in Table 2.

#### 3.6.3. Determining the User’s Gaze for Target Selection Based on Defuzzification Methods

**Figure 12:**
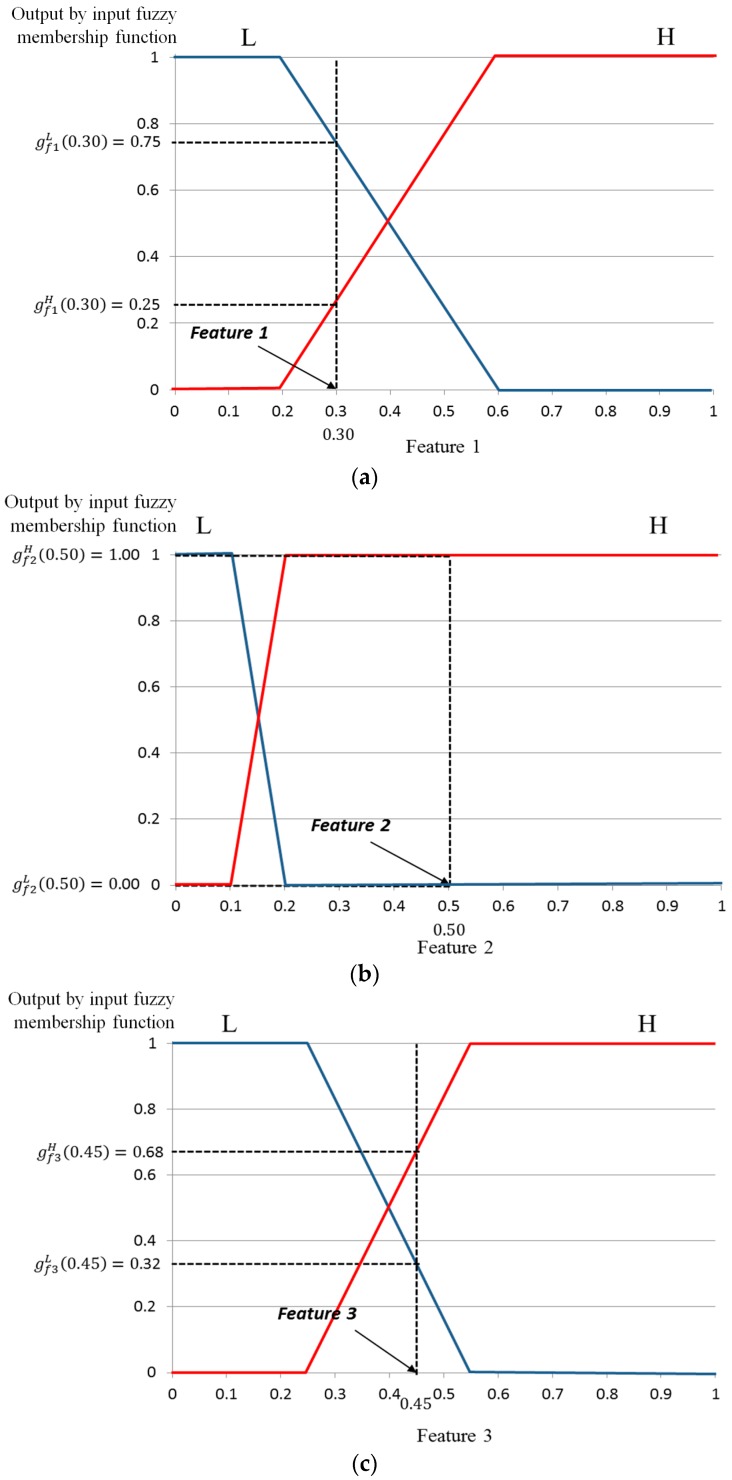
Finding the output value of the input membership function for three features: (**a**) feature 1; (**b**) feature 2; and (**c**) feature 3.

Using the three normalized input features, six corresponding values can be acquired using the input membership functions as shown in Figure 12. Three functions are defined as $g_{f1}$(·), $g_{f2}$(·), and $g_{f3}$(·). The corresponding output values of the three functions with input values of *f*1(feature 1)*, f*2(feature 2), and *f*3(feature 3) are denoted by$\;(g_{f1}^{L},\;g_{f1}^{H}),\;(g_{f2}^{L},$ $g_{f2}^{H})$, and $\left(g_{f3}^{L},g_{f3}^{H}\right)$. For example, suppose that the three input values for *f*1, *f*2, and *f*3 are 0.30, 0.50, and 0.45, respectively, as shown in Figure 12. The values of $(g_{f1}^{L},\;g_{f1}^{H}),\;(g_{f2}^{L},$ $g_{f2}^{H})$, and $\left(g_{f3}^{L},g_{f3}^{H}\right)$ are (0.75(L), 0.25(H)), (0.00(L), 1.00(H)), and (0.32(L), 0.68(H)), respectively, as shown in Figure 12. With these values, we can obtain the following eight combinations: (0.75(L), 0.00(L), 0.32(L)), (0.75(L), 0.00(L), 0.68(H)), (0.75(L), 1.00(H), 0.32(L)) …, and (0.25(H), 1.00(H), 0.68(H)).

The proposed method defines which of L and H can be used as inputs for the defuzzification step using the eight rules in Table 2. The MIN or MAX method is commonly used for this purpose. In the MIN method, the minimum value is selected from each combination set of three members and used as input for defuzzification. For the MAX method, the maximum value is selected and used as defuzzification input.

For example, for a combination set of (0.25(H), 0.00(L), 0.68(H)), the MIN method selects the minimum value (0.00) as input. For the MAX method, the maximum value (0.68) is selected. Then, on the basis of fuzzy logic rules from Table 2 (if H, L, and H, then M), values of 0.00(M) and 0.68(M) are finally determined by the MIN and MAX methods, respectively.

All values calculated by the MIN and MAX rules with eight combinations of this example are listed in Table 3. We refer to these values as “inference values” (IVs). As shown in Table 3, these IVs are used as inputs for defuzzification to obtain the output. The MIN and MAX rules were compared in our experiments.

**Figure 13:**
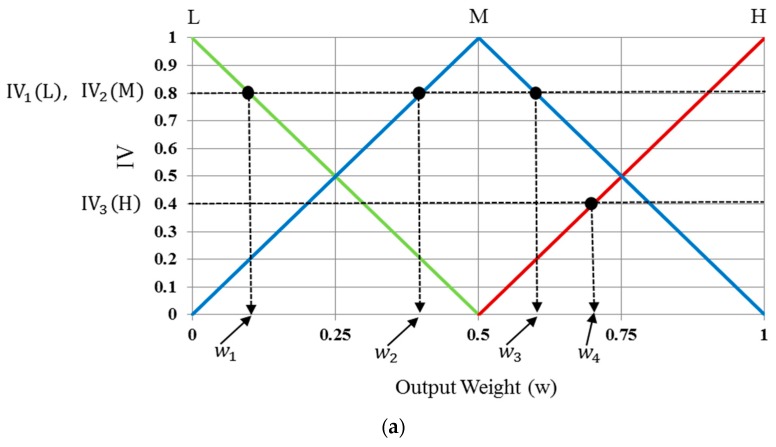

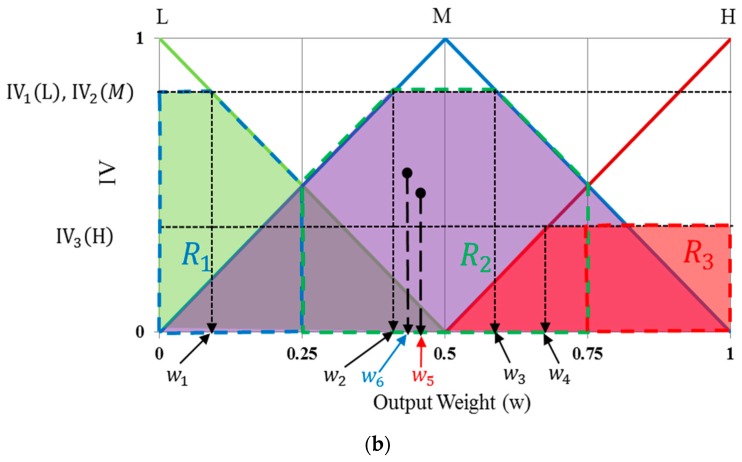
Obtaining output score values of fuzzy system using various defuzzification methods. (**a**) FOM, LOM, and MOM; (**b**) COG and BOA.

Several defuzzification methods are compared on fuzzy systems. We considered five of them, i.e., first of maxima (FOM), last of maxima (LOM), middle of maxima (MOM), center of gravity (COG), and bisector of area (BOA) [76,77,78,79]. In each defuzzification method excluding COG and BOA, the maximum values of IVs were used to calculate the output value. The maximum IVs were ${\mathrm{IV}}_{1}\left(L\right)\;\mathrm{and}$ ${\mathrm{IV}}_{2}\left(M\right)$, as shown in Figure 13a. In the FOM defuzzification method, as the name implies, the first value after defuzzification is selected as the optimal weight value, and is represented as $w_{1}$ in Figure 13a. The last defuzzification value, i.e., $w_{3}$ is the optimal weight value selected by the LOM method. The method that shows that the optimal weight value is obtained from the middle value of the optimal weight values of FOM and LOM is referred to as the MOM method, i.e., ($w_{\mathrm{MOM}}=\frac{1}{2}\left(w_{1}+w_{3}\right))$.

The output scores for the methods using the COG and the BOA are calculated differently from those of other defuzzification methods. The COG method is also known as center-of-area defuzzification. It calculates the output score based on the geometrical center of the non-overlapping regions, and is formed by the regions defined by all IVs. As shown in Figure 13b, regions *R*_1_, *R*_2_, and *R*_3_ are defined based on all IVs, where region *R*_1_ is the quadrangle defined by connecting four points (0, IV_1_(L)), (w_1_, IV_1_(L)), (0.5, 0), and (0, 0), *R*_2_ is the quadrangle defined by connecting four points (w_2_, IV_2_(M)), (w_3_, IV_2_(M)), (1, 0), and (0, 0), and *R*_3_ is that defined by connecting four points (w_4_, IV_3_(H)), (1, IV_3_(H)), (1, 0), and (0.5, 0). 

Finally, the optimal weight value of the fuzzy system ($w_{5}$) is calculated from the center of gravity of regions *R*_1_, *R*_2_, and *R*_3_ (considering the overlapping regions of *R*_1_, *R*_2_, and *R*_3_ only once), as shown in Figure 13b. The BOA is calculated by a vertical line ($w_{6}$) that divides the region defined by the IVs into two sub-regions of equal area, which is why it is called the bisector method. Following this, our fuzzy system determines that user gazes at the position for target selection, if the output score of the fuzzy system is greater than a threshold defined for the fuzzy system.

## 4. Experimental Results

**Figure 14:**
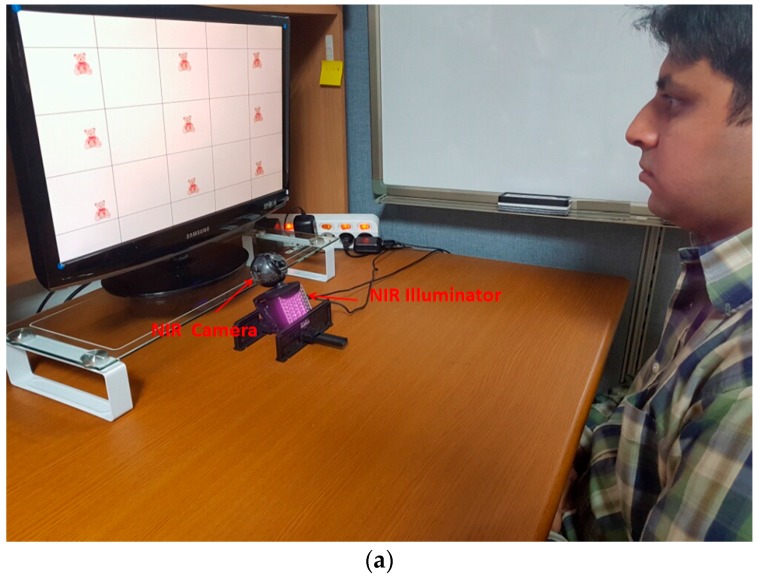

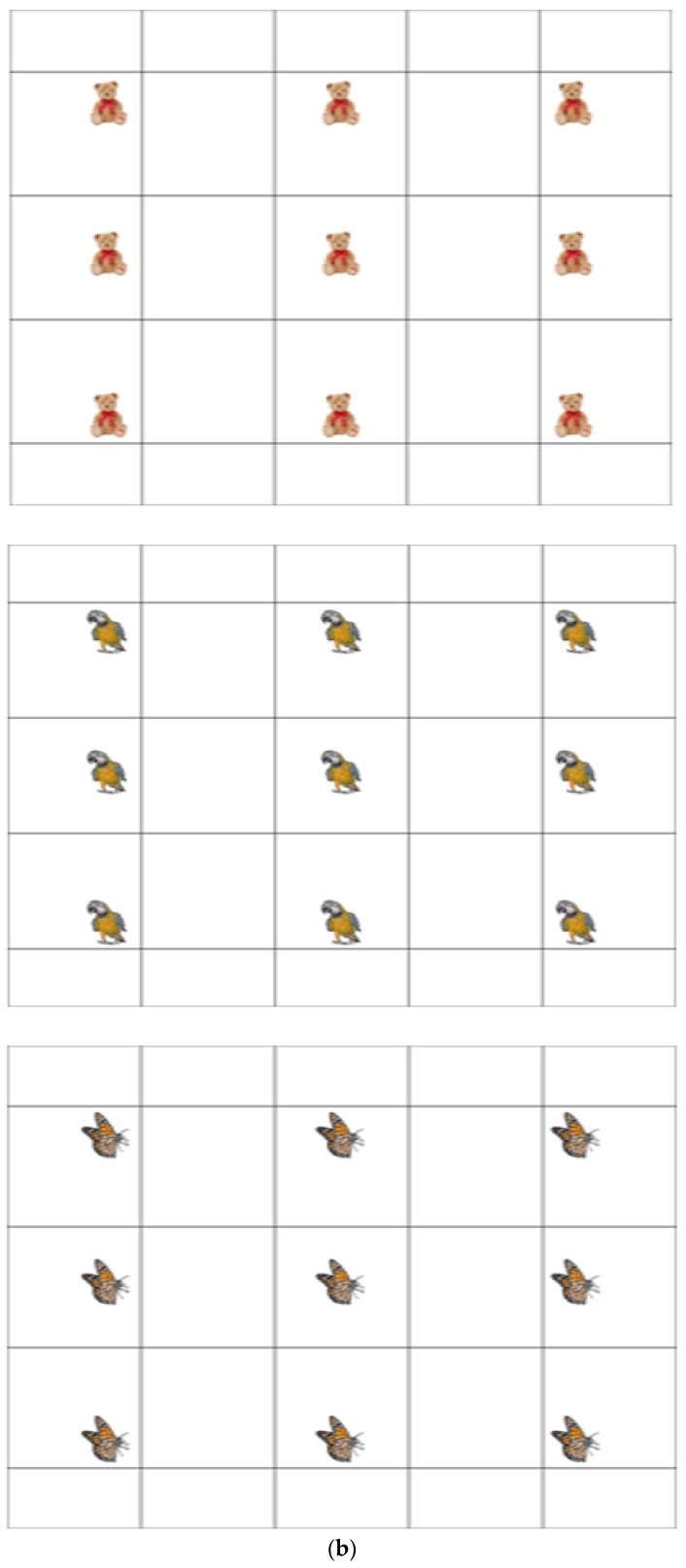
Experimental setup for proposed method. (**a**) Example of experimental environment; (**b**) Three screens of teddy bear (upper), bird (middle), and butterfly (lower) used in our experiments.

The performance of the proposed method for detecting user gaze for target selection was measured through experiments performed with 15 participants, where each participant attempted two trials for each of three target objects, i.e., a teddy bear, a bird, and a butterfly, displayed at nine positions on a 19-inch monitor, as shown in Figure 14. That is, three experiments were performed with these objects (teddy bear, bird, and butterfly) with each at nine positions on the screen. We collected 270 data items (15 participants × 2 trials × 9 gaze positions) for gazing for target selection, i.e., true positive (TP) data, and the same number of non-gazing data, i.e., true negative (TN) data, for each of the three experiments. Most participants were graduate students, and some of them were faculty or staff members of our university’s department. They were randomly selected by considering the variation in eye characteristics with age, gender, and nationality. All participants voluntarily participated in our experiments. Of the 15, five wore glasses and four people wore contact lenses. The remained 6 people did not wear glasses and contact lens. The ages of the participants ranged from 20 s to 40 s (mean age 29.3 years). Nine participants were male, and the other 6 ones were female. We made sure that participants of different nationalities were involved in our experiments: one Mongolian, one Tanzanian, two Pakistanis, four Vietnamese, and seven Koreans. Before the experiments, we provided the sufficient explanations of our experiments to all participants, and obtained informed (written) consents from all the participants.

**Figure 15:**
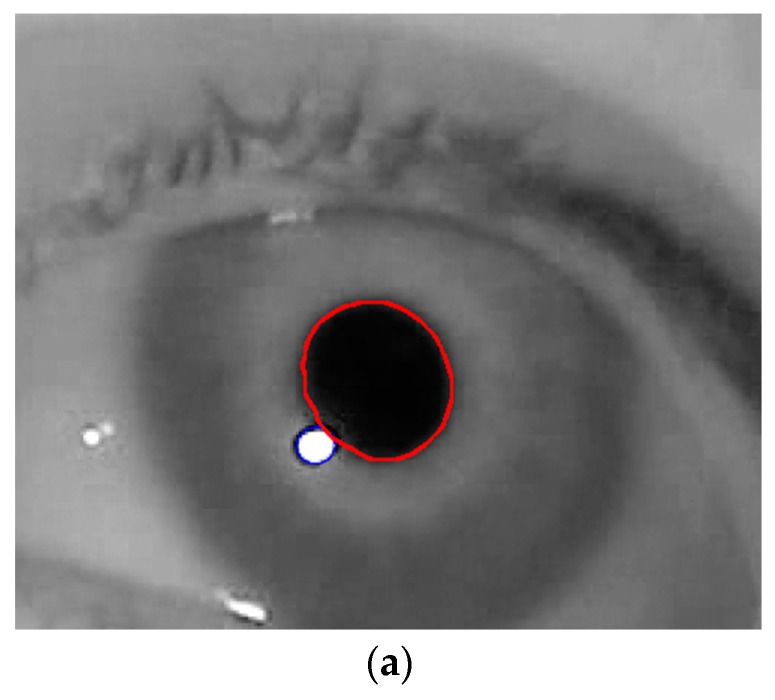

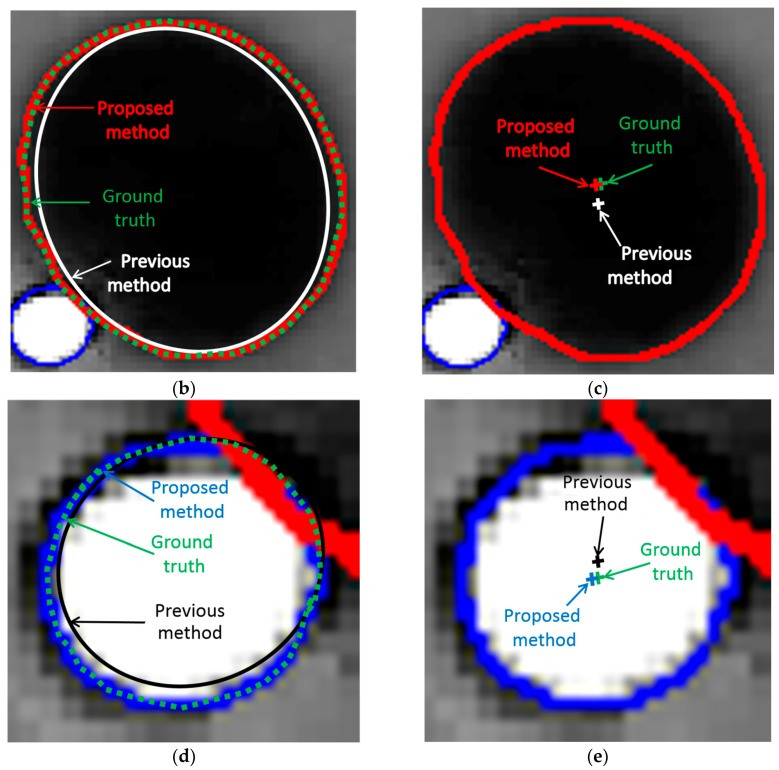
Comparative examples of detection of boundaries and centers of pupil and glint by our method, and the previous method and the ground truth. (**a**) Detected boundaries of pupil and glint in eye image; (**b**) Comparison of detection of boundaries of pupil; (**c**) Comparison of detection of center of pupil; (**d**) Comparison of detection of boundaries of glint; (**e**) Comparison of detection of center of glint.

In the first experiment, we compared the accuracy of our method, in detecting the boundary between the pupil and the glint as well as the center of each, with a previous method [24]. As shown in Figure 15b,c, the boundary and center of the pupil detected by our method were closer to the ground truth than those calculated by the previous method. Moreover, as shown in Figure 15d,e, the detected boundary and center of the glint according to our method were closer to the ground truth than the previous method. In this experiment, the boundary and center of the ground truth were manually chosen. Further, for all images, we measured detection errors based on Euclidean distance between the center of the ground truth and the center as detected by our method and the previous method [24]. As shown in Table 4, our method yielded a higher accuracy (lower error) in detecting the centers of the pupil and the glint than the previous method.

In the second experiment, the accuracy of the detection of the user’s gaze for target selection on TP and TN data, according to various defuzzification methods, was compared in terms of equal error rate (EER). Two types of errors, types I and II, were considered. The error of incorrectly classifying TP data as TN data was defined as a type I error, and that of incorrectly classifying TN data as TP data was defined as a type II error. As explained in the previous section, our system determines that the user’s gaze is employed (TP) if the output score of the fuzzy system is higher than the threshold. If not, our system determines that user’s gaze is not employed (TN). Therefore, type I and II errors occur depending on the threshold. If the threshold is increased, type 1 error increases and type II error decreases. With a smaller threshold, type I error decreases whereas type II error increases. When type I and II errors are most similar to the appropriate threshold, the EER is calculated by averaging the two errors.

**Experiment 1 (Bear)**

In the first experiment, a teddy bear was used as target object, as shown in Figure 14b. The classification results of TP and TN data according to the five defuzzification methods that used the MIN and MAX rules are listed in Table 5 and Table 6, respectively. As indicated in these tables, the smallest EER (approximately 0.19%) of classification was obtained by the COG with the MIN rule.

**Figure 16:**
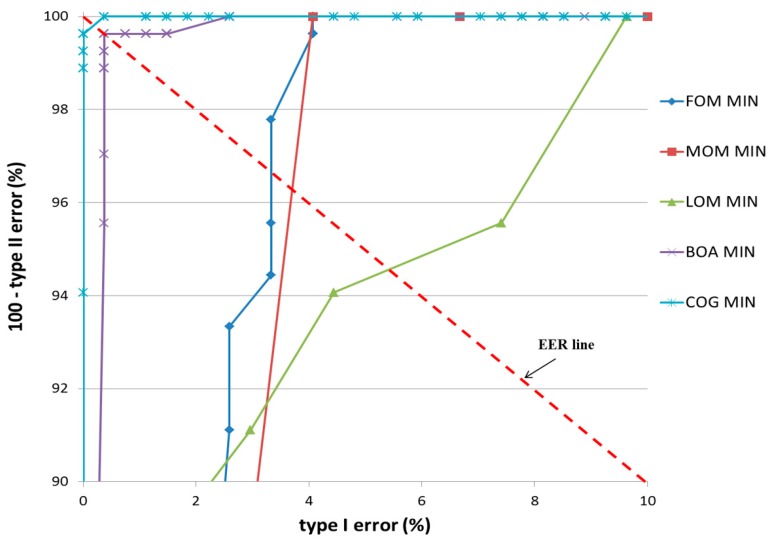
ROC curves of the classification results of TP and TN data according to different defuzzification methods with the MIN rule.

**Figure 17:**
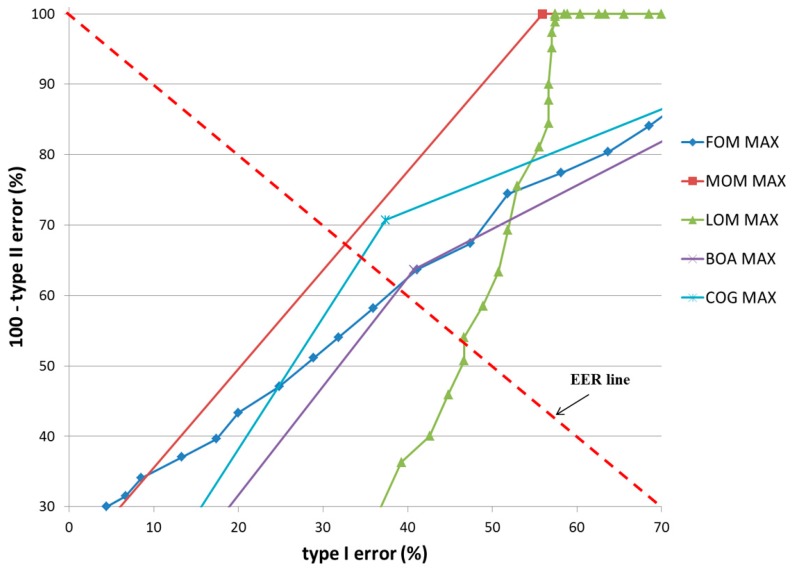
ROC curves of the classification results of TP and TN data according to different defuzzification methods with the MAX rule.

Figure 16 and Figure 17 show the receiver operating characteristic (ROC) curves for the classification results of TP and TN data according to the various defuzzification methods using MIN or MAX rules, respectively. The ROC curve represents the change in type I error (%) according to the increase in 100—type II error (%). 

In case that type I and II errors were small, the accuracy of that particular method was regarded as high. Therefore, if the ROC curve was closer to (0, 100) (“type I error” of 0% and “100—type II error” of 100%) on the graph, its accuracy was regarded as higher. As shown in these figures, the accuracy of classification by the COG with the MIN rule was higher than obtained by other defuzzification methods.

**Experiment 2 (Bird)**

In the second experiment, a bird was used as target object as shown in Figure 14b. The classification results of TP and TN data according to the five defuzzification methods that used the MIN and MAX rules are listed in Table 7 and Table 8, respectively. As indicated in these tables, the smallest EER (approximately 0%) of classification was obtained by the COG with the MIN rule.

**Figure 18:**
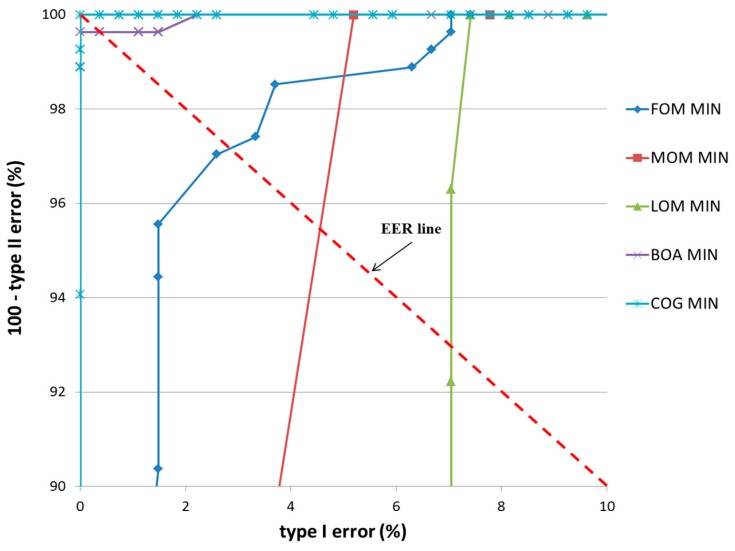
ROC curves of the classification results of TP and TN data according to different defuzzification methods with the MIN rule.

**Figure 19:**
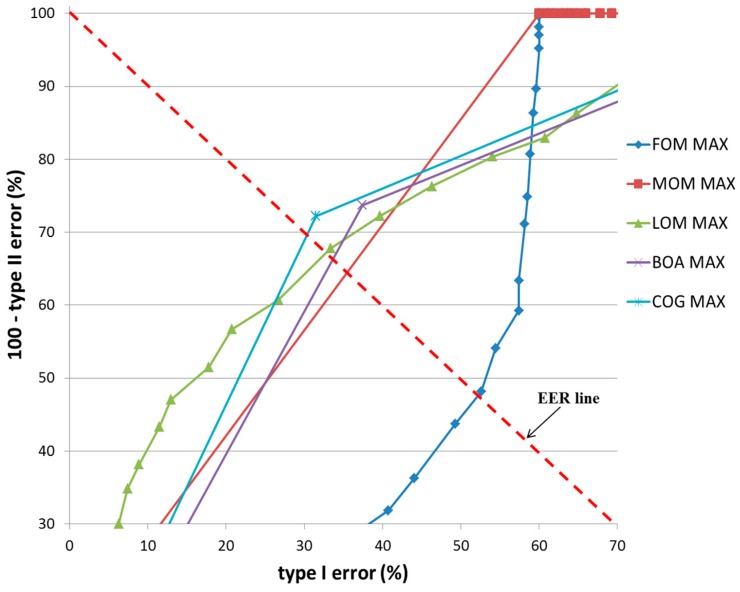
ROC curves of the classification results of TP and TN data according to different defuzzification methods with the MAX rule.

Figure 18 and Figure 19 show the ROC curves for the classification results of TP and TN data according to the various defuzzification methods using MIN or MAX rules. As is shown, the accuracy of classification by the COG with the MIN rule was higher than for other defuzzification methods.

**Experiment 3 (Butterfly)**

In the third experiment, a butterfly was used as target object as shown in Figure 14b. The classification results of TP and TN data according to the five defuzzification methods that used the MIN and MAX rules are listed in Table 9 and Table 10, respectively. As indicated in these tables, the smallest EER (approximately 0.19%) of classification was obtained by the COG with the MIN rule.

**Figure 20:**
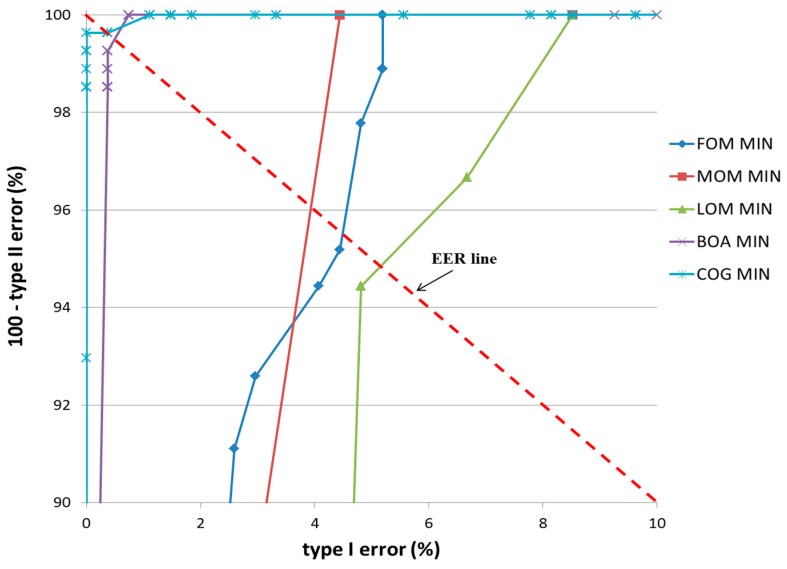
ROC curves of the classification results of TP and TN data according to different defuzzification methods with the MIN rule.

**Figure 21:**
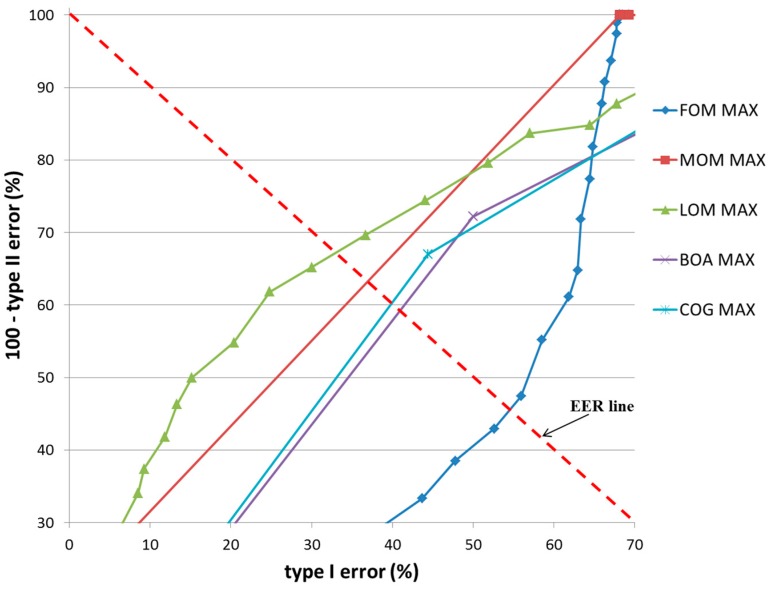
ROC curves of the classification results of TP and TN data according to different defuzzification methods with the MAX rule.

Figure 20 and Figure 21 show the ROC curves for the classification results of TP and TN data according to the various defuzzification methods using the MIN or MAX rules. As is shown, the accuracy of classification by the COG with the MIN rule was higher than by other defuzzification methods.

The above results were verified by comparing the proposed method using three features (change of pupil size w.r.t. time by template matching, change in gaze position within short dwell time, and the texture information of monitor image at gaze target) with the previous method [24] using two features (change of pupil size w.r.t. time by peakedness, and change in gaze position within short dwell time). In addition, we compared the accuracy by proposed method with that by using three features (change of pupil size w.r.t. time by peakedness, change in gaze position within short dwell time, and the texture information of monitor image at gaze target). For convenience, we call the last method “Method A”. The only difference between the proposed method and “Method A” is that change in pupil size w.r.t. time is measured by template matching in proposed method but by peakedness in “Method A”.

**Figure 22:**
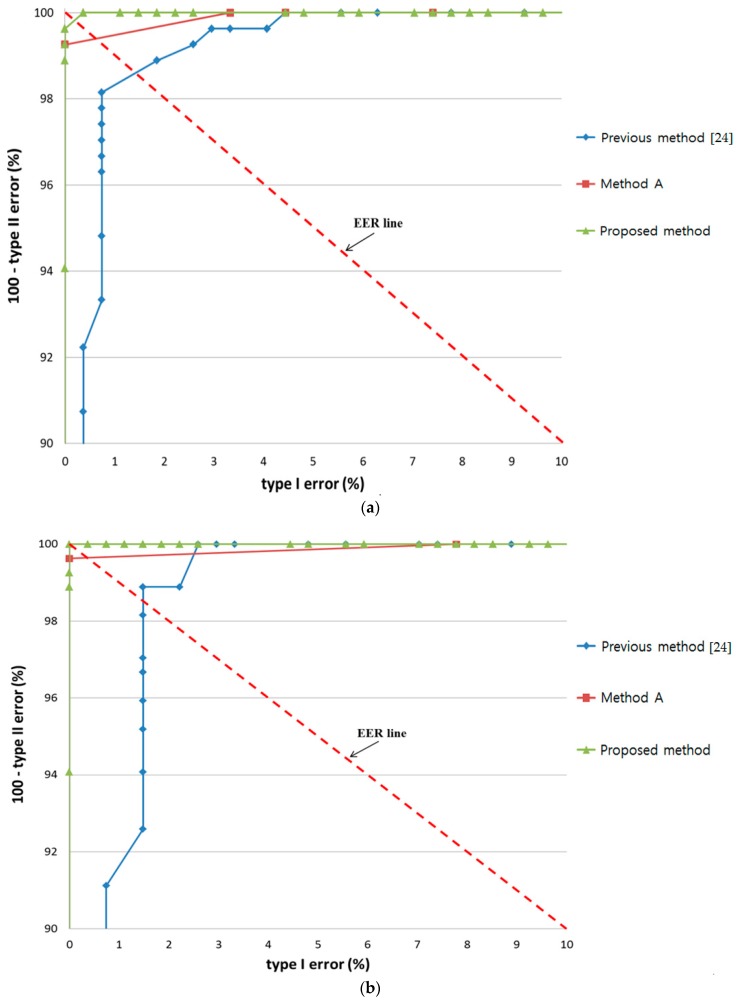

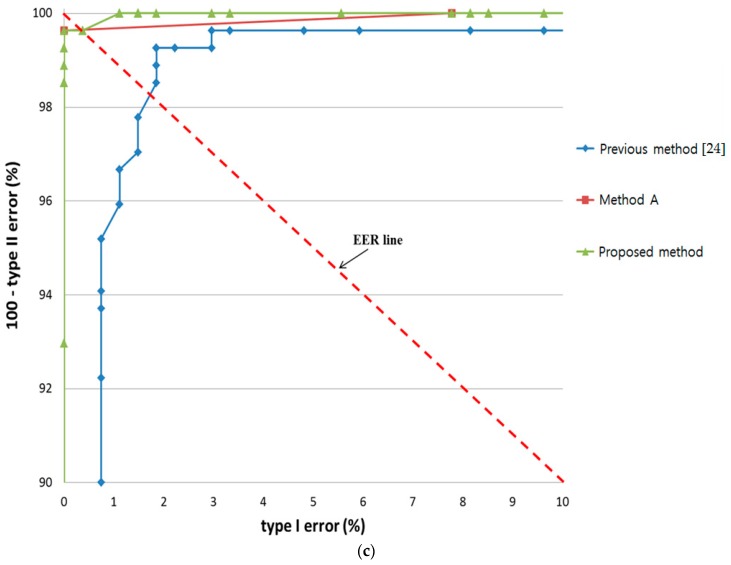
Comparison of results by proposed method with the results by “Method A” and previous method with experiments involving (**a**) bear; (**b**) bird; and (**c**) butterfly.

In all cases, the ROC curves of the highest accuracy among the various defuzzification methods with the MIN or MAX rules are shown. The accuracy of by proposed method was always higher than that of “Method A” and the previous method [24] in all cases of the three experiments, i.e., bear, bird, and butterfly, as shown in Figure 22.

In the next experiment, we compared the accuracy of proposed method, “Method A”, and the previous method [24] when noise was included in the input data. All features of the proposed and the previous method was affected by the accurate detection of pupil size and gaze position, which were in turn affected by the performance of the gaze tracking system. Thus, we included Gaussian random noises in the detected pupil size and gaze position. 

**Figure 23:**
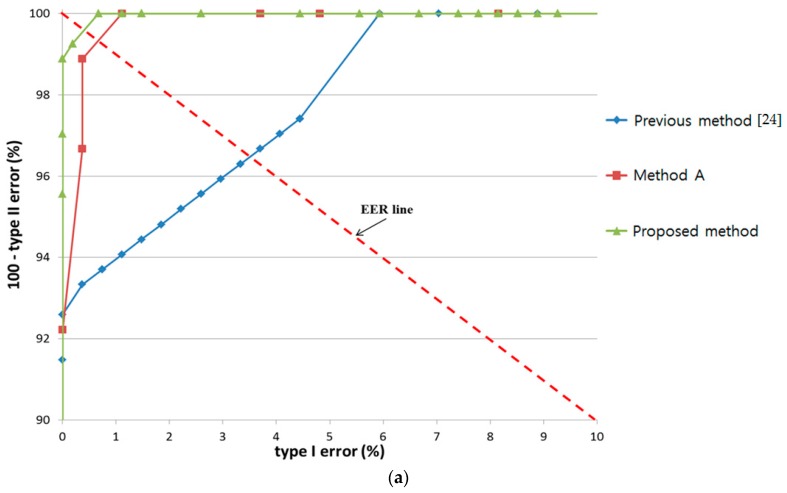

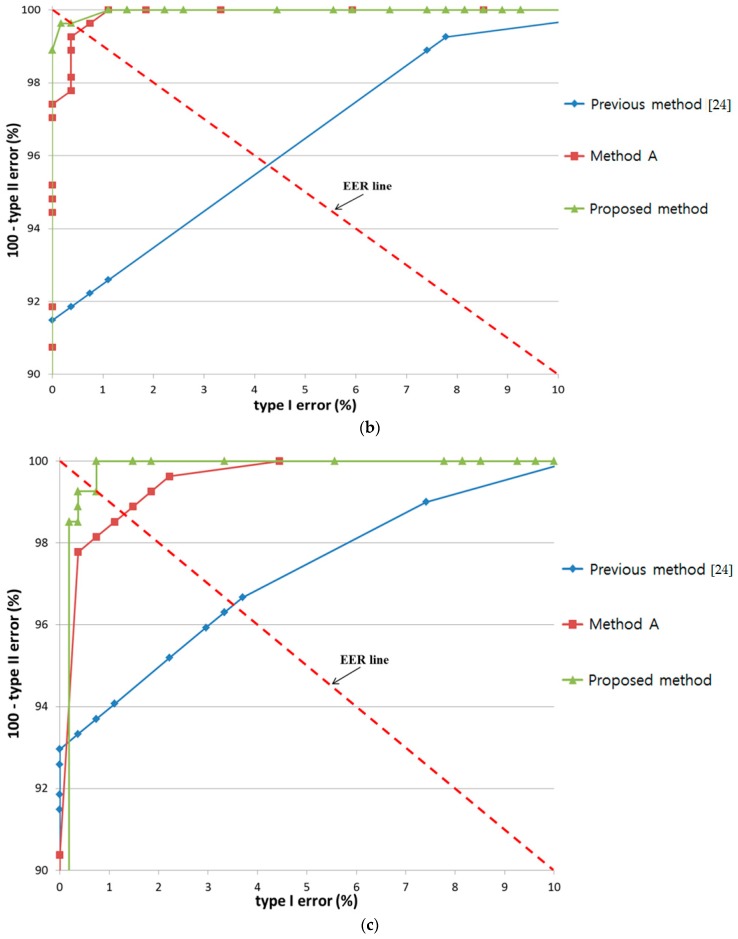
Effect of noise on results by proposed method with the results by “Method A” and previous method with experiments involving (**a**) bear; (**b**) bird; and (**c**) butterfly.

As shown in Figure 23, noise had a stronger effect on the previous method [24] and “Method A” than the proposed method in all three experiments. A notable decrease in accuracy with increase in EER was observed in the latter two methods when noise caused incorrect detection of pupil size and gaze.

In the next experiments, we compared the usability of our system with the conventional dwell time-based selection method [13,14]. We requested the 15 participants to rate the level of convenience and interest in performing the target selection task of the proposed method and a dwell time-based method by using a questionnaire (5: very convenient, 4: convenient, 3: normal, 2: inconvenient, 1: very inconvenient, in case of a convenience questionnaire) (5: very interesting, 4: interesting, 3: normal, 2: uninteresting, 1: very uninteresting, in case of an interest questionnaire). In order to render our results unaffected by participant learning and physiological state, such as fatigue, we provided a rest time of 10 minutes to each participant between experiments. Based on [13,14], dwell time for target selection was set at 500 ms. That is, when the change in the user’s gaze position for our feature 2 was lower than the threshold, and this state was maintained for longer than 500 ms, target selection was activated.

**Figure 24:**
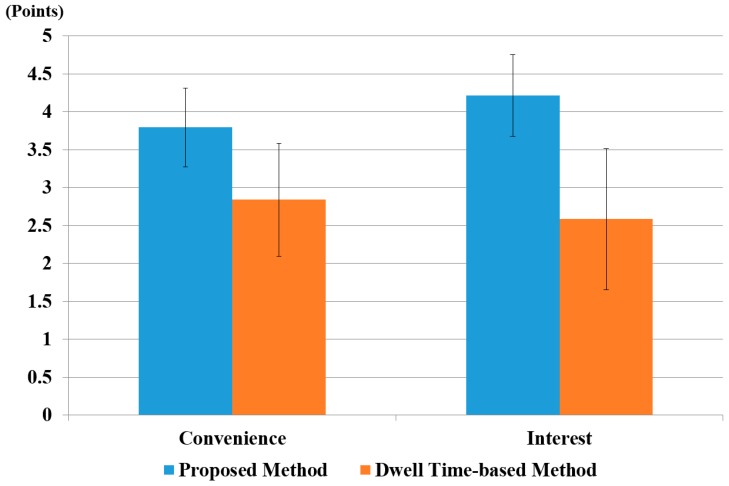
The comparative results of subjective tests of 15 users using the dwell time-based method and the proposed method.

The average scores are shown in Figure 24, which shows that our method scored higher than the conventional dwell time-based method in terms of both convenience and interest.

We also performed a *t*-test [80] to prove that user’s convenience on the proposed method was statistically higher than that of the conventional dwell time-based method. The *t*-test was performed by taking two independent samples of data: user’s convenience on our system (*µ* = 3.8, *σ* = 0.5) and with the conventional dwell time-based method (*µ* = 2.8, *σ* = 0.7). The calculated *p*-value was approximately 3.4 × 10^−4^, which was smaller than the 99% (0.01) significance level. Hence, the null hypothesis for the *t*-test, i.e., no difference between the two independent samples, was rejected. Therefore, we can conclude that there was a significant difference, up to 99%, in user convenience between our proposed method and the dwell time-based method.

A *t*-test analysis based on user interest was also performed. This also yielded similar results, i.e., user interest on our proposed system (*µ* = 4.2, *σ* = 0.5) was higher than with the dwell time-based system (*µ* = 2.6, *σ* = 0.9). The calculated *p*-value was 6.74 × 10^−6^, i.e., smaller than the significance level of 99% (0.01). Therefore, we concluded a significant difference in user interest between our system and the dwell time-based system. The average score for convenience and interest was higher for the proposed method because it is more natural than a conventional dwell time-based method.

In order to analyze the effect of the difference in size between two groups, we performed Cohen’s *d* analysis [81]. It classifies the difference as small if it is within the range 0.2–0.3, medium if it is 0.5, and large if it is greater than or equal to 0.8. We calculated the value of Cohen’s *d* for convenience and interest. For user convenience, it was 1.49, which was in the large-effect category; hence, it had a major effect on the difference between the two groups. For user interest, the calculated Cohen’s *d* was approximately 2.14, which was also in the large-effect category. Hence, we concluded that user convenience and user interest showed a large effect in the difference between our proposed method and dwell time-based method. 

**Figure 25:**
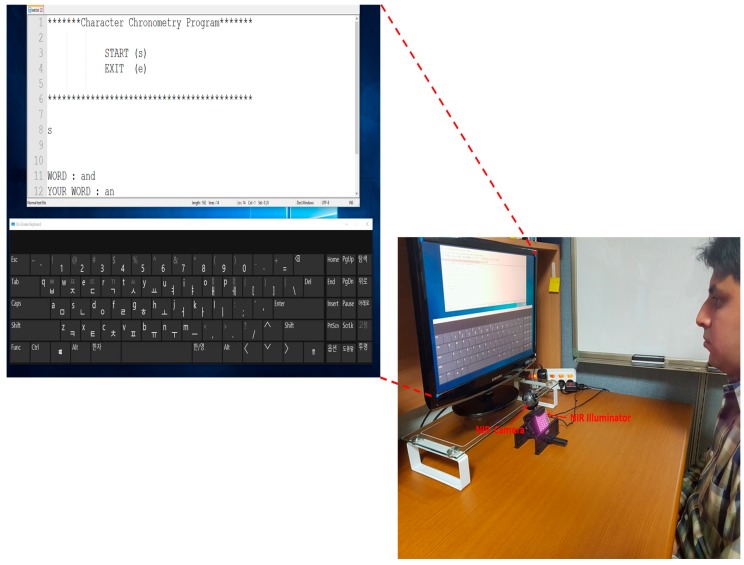
Example of experiment where a user types words on our system.

To confirm the practicality of our method, we performed additional experiments using an on-screen keyboard based on our method, where each person typed a word through our system on an on-screen keyboard displayed on a monitor, as shown in Figure 25. All 15 subjects from before participated in the experiments, and a monitor with a 19-inch screen and resolution of 1680 × 1050 pixels was used. Twenty sample words were selected based on frequency of use as shown in Table 11 [82]. As shown in Table 11, the left-upper words “the” and “and” were more frequently used than the right-lower words “all” and “would”. If the user’s gaze detected by our method was associated with a specific button, and our method had determined the user’s gaze, the corresponding character was selected and displayed, as shown in Figure 25.

**Figure 26:**
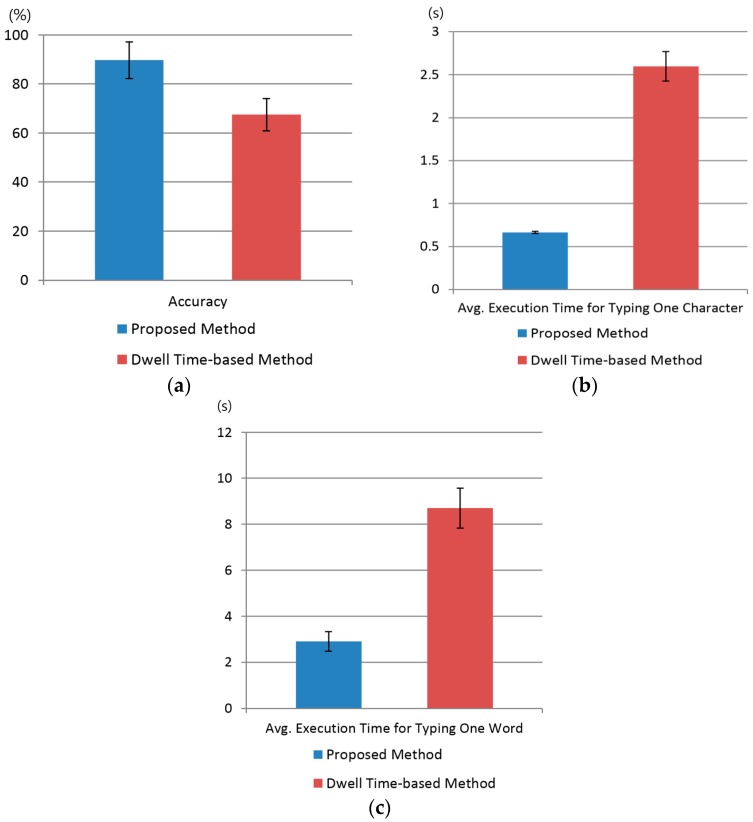
The comparative results of proposed method with dwell time-based method using virtual keyboard in terms of accuracy and execution time. (**a**) Accuracy; (**b**) Average execution time for typing one character; (**c**) Average execution time for typing one word.

**Figure 27:**
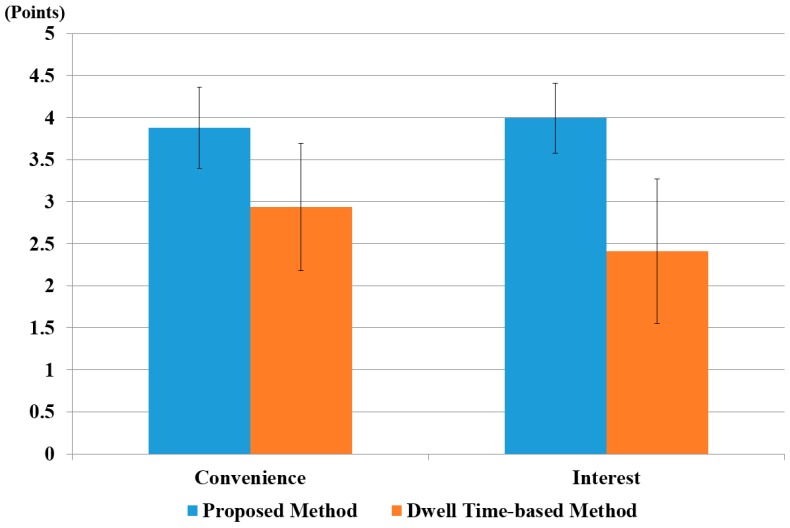
The comparative results of proposed method, with the dwell time-based method using virtual keyboard in terms of convenience and interest.

We performed a *t*-test to prove that our method is statistically better than the conventional dwell time-based method [13,14], as shown in Figure 26 and Figure 27. In order to immunize our results against participant learning and physiological state, such as fatigue, we gave a 10-min rest to each participant between experiments. Based on [13,14], dwell time for target selection was set at 500 ms. In all cases, our gaze detection method was used for fair comparison, and selection was performed using our method or the dwell time-based method. We conducted our statistical analysis by using four performance criteria, i.e., accuracy, execution time, interest, and convenience.

As shown in Figure 26a, the *t*-test was performed using two samples independent of each other: the user’s typing accuracy using our system (*µ* = 89.7, *σ* = 7.4) and the conventional dwell time-based method (*µ* = 67.5, *σ* = 6.5). The calculated *p*-value was approximately 1.72 × 10^−9^, smaller than for a 99% (0.01) significance level. Hence, the null hypothesis for the *t*-test, i.e., no difference between two independent samples, was rejected. Therefore, we concluded that there was a significant difference of up to 99% in accuracy between the proposed method and the dwell time-based method.

Similarly, a *t*-test analysis based on average execution time for typing one character was performed, as shown in Figure 26b, this test yielded similar results, i.e., the average execution time for typing one character with our system (*µ* = 0.67, *σ* = 0.013) and the dwell time-based system (*µ* = 2.6, *σ* = 0.17). The calculated *p*-value was 2.36 × 10^−16^, i.e., smaller than the significance level of 99% (0.01). Therefore, we concluded that there was a significant difference in execution time for typing one character on a virtual keyboard between our system and the dwell time-based system. Although dwell time for target selection was set at 500 ms in the dwell-time-based method, it was often case that the participant did not wait for this long and move his or her gaze position, which increased the average execution time for typing one character, as shown in Figure 26b.

A *t*-test analysis based on execution time for typing one word was also conducted as shown in Figure 26c. We compared our proposed system (*µ* = 2.9, *σ* = 0.42) with the dwell time-based system (*µ* = 8.7, *σ* = 0.87). The calculated *p*-value was 5.7 × 10^−16^, i.e., smaller than the significance level of 99% (0.01). Therefore, we concluded that there was a significant difference between the execution time for typing one word on a virtual keyboard between our system and the dwell time-based system.

As shown in Figure 27, the *t*-test analysis for user interest compared our proposed system (*µ* = 4.0, *σ* = 0.4) with the dwell time-based system (*µ* = 2.4, *σ* = 0.9). The calculated *p*-value was 6.1 × 10^−7^, i.e., smaller than the significance level of 99% (0.01). Therefore, we concluded that there was a significant difference in user interest between our system and the dwell time-based system.

Similarly, we performed a *t*-test with respect to user convenience. We noted that with our system (*µ* = 3.9, *σ* = 0.5) and the conventional dwell time-based method (*µ* = 2.9, *σ* = 0.8), the calculated *p*-value was approximately 3.4 × 10^−4^, which was smaller than the 99% (0.01) significance level. Hence, the null hypothesis for the *t*-test, i.e., no difference between two independent samples was rejected. Therefore, we concluded a significant difference up to 99% in user convenience with our proposed method and the dwell time-based method. As shown in Figure 27, the average score for convenience and interest was higher for the proposed method because it is more natural than a conventional dwell time-based method.

Similarly, we calculated the value of Cohen’s *d* for convenience, interest, average execution time for typing, and accuracy. For user convenience and interest, they were 1.49 and 2.34, respectively, and lay in a large category. Hence, this had a significant effect on the difference between the two groups. For average execution times for one character, one word, and accuracy, we calculated the value of Cohen’s *d* as approximately 15.92, 8.51, and 3.19, respectively, which also lay in the large-effect category. Hence, from the *p* value and Cohen’s *d*, we concluded that user convenience, interest, average execution time, and accuracy were significantly different for the proposed and the dwell time-based methods [13,14]. 

In our experiment, the screen resolution is 1680 × 1050 pixels on a 19-inch monitor. The z-distance between the monitor and user’s eye ranges 60 to 70 cm. Considering the accuracy (about ±1°) of gaze detection in our system, the minimum distance between two objects on the monitor screen should be about 2.44 cm (70 cm × tan(2°)) which corresponds to approximately 82 pixels.

## 5. Conclusions

In this study, we proposed a method for detecting the user’s gaze for target selection using a gaze tracking system based on an NIR illuminator and a camera. The pupil center with the boundary and the glint center were more accurately based on an enhanced Chan–Vese algorithm. We employed three features—change of pupil size w.r.t. time measured by template matching, change in gaze position within short dwell time, and the texture information of monitor image at gaze target. These features as input values were combined with a fuzzy system where optimal input membership functions were designed based on the maximum entropy criterion and the user’s gaze for target selection was determined through defuzzification methods. Performance was evaluated by comparing the defuzzification results with the EER and ROC curves. We verified from the results that the COG method using the MIN rule is suitable in terms of accuracy for different objects with varying amounts of texture. In future work, we will investigate a method to enhance performance by combining our proposed features with various physiological ones, such as electrocardiography (ECG) data, electroencephalogram (EEGs) data, or skin temperature (SKT).

## Figures and Tables

**Table 1:** Comparison of previous and proposed methods.

Category	Method	Advantages	Disadvantages
Gaze-based methods	Use a single modality, i.e., eye blink [9,22], dwell time [12,13,14], antisaccades [15], on and off screen buttons [16], key strokes [19,20], face frowning [21], eye brow raises [22], and smiling [23]	- Single sensing modality is used, and so is simpler in design compared to the method based on multiple modalities - Fewer input data items are required compared to multiple modalities	- Lower accuracy of selection of target than methods based on multiple modalities - Completely dependent on a single modality; minor errors in sensing data can badly affect overall results - Some methods using a single modality are not feasible for users with high levels of motor disabilities, especially for those who can only move their eyes [19,20,21,22,23]
	Use multiple modalities, i.e., pupil accommodation and dwell time [24]	- Accuracy of intentional object selection is high compared to methods based on single modality	- There is room for further enhancement of detecting user’s gaze for target selection - Incorrect detection of pupil size and the centers of pupil and corneal glint can significantly affect accuracy
Visual saliency-based methods	Bottom-up approach [39,40,41,42,43,44,45]	- Performs well for detecting low-level features	- Semantic information is not considered
	Top-down approach [46,47,48,49]	- Performs well for high-level features	- Performance can be affected by the correct detection of high-level features, i.e., face detection
	Learning-based method [50,51,52,53,54,55,56,57]	- Efficient for complex input data	- Performs well for particular category of data on which it is trained
Fusing gaze-based method with visual saliency-based method	Combining pupil accommodation with template matching, short dwell time, and the texture information of visual saliency by fuzzy system (**proposed method**)	- Higher accuracy of target selection - Less affected by the incorrect detection of pupil and corneal glint	- More input data needs to be processed compared to gaze-based and visual saliency-based methods

**Table 2:** Fuzzy rules based on features 1–3.

Feature 1	Feature 2	Feature 3	Output of Fuzzy System
L	L	L	L
L	L	H	L
L	H	L	M
L	H	H	H
H	L	L	L
H	L	H	M
H	H	L	H
H	H	H	H

**Table 3:** IVs obtained with eight combinations.

Feature 1	Feature 2	Feature 3	IV	
			MIN Rule	MAX Rule
0.75(L)	0.00(L)	0.32(L)	0.00(L)	0.75(L)
0.75(L)	0.00(L)	0.68(H)	0.00(L)	0.75(L)
0.75(L)	1.00(H)	0.32(L)	0.32(M)	1.00(M)
0.75(L)	1.00(H)	0.68(H)	0.68(H)	1.00(H)
0.25(H)	0.00(L)	0.32(L)	0.00(L)	0.32(L)
0.25(H)	0.00(L)	0.68(H)	0.00(M)	0.68(M)
0.25(H)	1.00(H)	0.32(L)	0.25(H)	1.00(H)
0.25(H)	1.00(H)	0.68(H)	0.25(H)	1.00(H)

**Table 4:** Average Euclidean distance between the center of the ground truth the detected center by our method and previous method (unit: pixels).

Pupil/Glint Center	Method	Average Euclidean Distance
Pupil Center	Proposed method	2.1
	Previous method [24]	4.6
Glint Center	Proposed method	2.5
	Previous method [24]	4.7

**Table 5:** Type I and II errors with EER using the MIN rule (unit: %).

Defuzzification Method	Threshold	No. of Type I Errors	No. of Type II Errors	EER
FOM	0.36	9	6	2.78
MOM	0.50	11	0	2.04
LOM	0.73	12	16	5.19
BOA	0.27	1	1	0.37
COG	0.30	0	1	0.19

**Table 6:** Type I and II errors with EER using the MAX rule (unit: %).

Defuzzification Method	Threshold	No. of Type I Errors	No. of Type II Errors	EER
FOM	0.09	111	98	38.7
MOM	0.50	151	0	27.96
LOM	0.89	126	124	46.3
BOA	0.50	110	98	38.52
COG	0.50	101	79	33.33

**Table 7:** Type I and II errors with EER using the MIN rule (unit: %).

Defuzzification Method	Threshold	No. of Type I Errors	No. of Type II Errors	EER
FOM	0.26	7	8	2.78
MOM	0.50	14	0	2.60
LOM	0.60	19	21	7.41
BOA	0.25	0	1	0.19
COG	0.27	0	0	0

**Table 8:** Type I and II errors with EER using the MAX rule (unit: %).

Defuzzification Method	Threshold	No. of Type I Errors	No. of Type II Errors	EER
FOM	0.11	142	140	52.22
MOM	0.50	162	0	30
LOM	0.91	90	87	32.78
BOA	0.50	101	71	31.85
COG	0.50	85	75	29.63

**Table 9:** Type I and II errors with EER using the MIN rule (unit: %).

Defuzzification Method	Threshold	No. of Type I Errors	No. of Type II Errors	EER
FOM	0.35	12	13	4.63
MOM	0.47	12	0	2.22
LOM	0.71	13	15	5.19
BOA	0.37	2	0	0.37
COG	0.30	0	1	0.19

**Table 10:** Type I and II errors with EER using the MAX rule (unit: %).

Defuzzification Method	Threshold	No. of Type I Errors	No. of Type II Errors	EER
FOM	0.11	151	142	54.26
MOM	0.50	184	0	34.07
LOM	0.90	81	94	32.41
BOA	0.50	135	75	38.89
COG	0.50	120	89	38.7

**Table 11:** Twenty sample words used for experiments.

the, and, that, have, for, not, with, you, this, but, his, from, they, say, her, she, will, one, all, would
